# Multiple crosslinked, self-healing, and shape-adaptable hydrogel laden with pain-relieving chitosan@borneol nanoparticles for infected burn wound healing

**DOI:** 10.7150/thno.102569

**Published:** 2025-01-02

**Authors:** Zexing Deng, Yi Guo, Xuefeng Wang, Jiajie Song, Guan Yang, Litong Shen, Yuheng Wang, Xin Zhao, Baolin Guo, Wen Wang

**Affiliations:** 1 Department of Radiology, Functional and Molecular Imaging Key Lab of Shaanxi Province, Tangdu Hospital, Air Force Medical University, Xi'an, 710038, Shaanxi, China.; 2 College of Materials Science and Engineering, Xi'an University of Science and Technology, Xi'an, 710054, China.; 3 Shaanxi Key Laboratory of Brain Disorders, Institute of Basic and Translational Medicine, Xi'an Medical University, Xi'an, 710021, China.; 4 Pilot Selection Bureau of PLA Air Force, Beijing, 100195, China.; 5 State Key Laboratory for Mechanical Behavior of Materials, and Frontier Institute of Science and Technology, Xi'an Jiaotong University, Xi'an, 710049, China.; 6 Key Laboratory of Shaanxi Province for Craniofacial Precision Medicine Research, College of Stomatology, Xi'an Jiaotong University, Xi'an, 710049, China.

**Keywords:** nanoscale borneol, pain relief, antibacterial and anti-inflammatory hydrogel, self-healing and self-adaptable hydrogel, infected burn wound

## Abstract

*Rationale:* Next-generation wound dressings with multiple biological functions hold promise for addressing the complications and pain associated with burn wounds. *Methods:* A hydrogel wound dressing loaded with a pain-relieving drug was developed for treating infected burn wounds. Polyvinyl alcohol chemically grafted with gallic acid (PVA-GA), sodium alginate chemically grafted with 3-aminobenzeneboronic acid (SA-PBA), Zn^2+^, and chitosan-coated borneol nanoparticles with anti-inflammatory and pain-relieving activities were combined to afford a nanoparticle-loaded hydrogel with a PVA-GA/Zn^2+^/SA-PBA network crosslinked via multiple physicochemical interactions. *Results:* The developed hydrogel demonstrated adhesiveness, self-healing, shape adaptability, injectability, degradability, conformity to complicated wound surfaces, and other desirable biological functions, including a pH-responsive drug release behavior and antibacterial, antioxidant, anti-inflammatory, and proangiogenic activities. In a murine scald wound model, the hydrogel effectively prevented infection by *Staphylococcus aureus* and downregulated pain perception (measured using mouse grimace scale scores and hind paw lifting and licking times), thereby accelerating wound healing. *Conclusion:* This study provides broad prospects for the development of new hydrogel systems that can substantially improve the dynamic management of infected burn wounds.

## Introduction

The skin, which has the largest surface area of all organs, provides protection but is susceptible to external damage, such as cuts, burns, and scratch injuries 1-3. Burn wounds, generally formed upon exposure to excessive heat or chemicals, cause hundreds of thousands of deaths annually 4, 5. They are characterized by prolonged healing times, sustained severe burning sensations, irregular shapes, and susceptibility to bacterial infections 6, 7. Thus, burn wound treatment is a widespread challenge in clinical practice 8, 9. Auto-skin transplantation, the gold standard of burn wound therapy 10, is often limited by the insufficient skin availability and pain. Therefore, intelligent multifunctional wound dressings, including films 11, foams 12, elastomers 13, nanofibers 14, and hydrogels 15, have been developed for burn wound healing. Among them, hydrogels, usually comprising hydrophilic polymers and copious amounts of water 16, are ideally suited for this purpose because they provide a moist environment with antibacterial, antioxidant, anti-inflammatory, and wound exudate absorbing properties while enabling wound monitoring and exhibiting shape adaptability. In some studies, these advantages have been integrated into a single hydrogel platform for healing infected burn wounds 17-22. However, the engineering of bioactive (e.g., pain-easing) hydrogel dressings to cope with infected burn wounds remains challenging.

Pain relief is an important aspect of burn wound treatment. Borneol, a traditional Chinese medicine, features cooling, pain-easing, antibacterial, and anti-inflammatory properties and has been used as a drug additive 23, causing fewer side effects than pain-attenuating (e.g., opioid) drugs. However, the poor water solubility and insufficient bioavailability of borneol limit its applications in wound healing, which highlights the need to improve these parameters through borneol modification. Chitosan (CS) is a natural pH-responsive antibacterial polymer broadly used in wound healing 24, 25. It will show great promising to incorporate CS-coated borneol (CS@BN) nanoparticles into a multifunctional hydrogel matrix to afford a dressing for healing infected burn wounds.

Burn wounds are susceptible to repeated bacterial (e.g., *Staphylococcus aureus* and* Pseudomonas aeruginosa*) infections 26, 27, which delay epidermis formation, vascularization, and other healing processes and can lead to various complications, including acidic wound environments, aggravation of inflammation, and even sepsis 28, 29. Owing to its polyphenolic nature, gallic acid (GA) exhibits antibacterial and antioxidant properties and can chelate metal ions (e.g., Mg^2+^ and Zn^2+^) to form networks 30. These metal ions, including Zn^2+^, can have pivotal biological functions, as exemplified by antibacterial and angiogenesis-promoting effects, and accelerate wound healing 31, 32.

Self-healing hydrogels are primarily prepared through the introduction of dynamic covalent bonds (e.g., boronic ester bonds 33, 34 and bonds formed via Schiff-base reactions 35) and/or physical interactions (e.g., hydrogen bonds 36, ion coordination/interactions 37, and host-guest interactions 38). Boronic ester bonds are typical dynamic covalent bonds used to build self-healing hydrogels. However, hydrogels crosslinked with one type of dynamic covalent bonds or physical noncovalent bonds may exhibit low mechanical strength and self-healing efficiency. Hence, double or multiple crosslinking mechanisms have been used in the design of self-healing hydrogels with enhanced mechanical strength and self-healing efficiency to treat dynamic irregular burn wounds 39, 40. Polyvinyl alcohol (PVA), a Food and Drug Administration approved biocompatible and degradable polymer with abundant hydroxyl groups, has been used to produce hydrogel dressings 41. The decoration of PVA chains with GA can improve the antioxidant and adhesive properties of the former because of the polyphenolic nature of the latter. Sodium alginate (SA) is another typical natural biodegradable polymer that can chelate metal ions, including Ca^2+^, Zn^2+^, and Sr^2+^, to form a hydrogel network 42, 43. Therefore, considerable attention has been drawn to the development of physicochemically crosslinked injectable hydrogels with adhesive, self-healing, biodegradable, shape-adaptable, pain-attenuating, antibacterial, antioxidant, anti-inflammatory, and vascularization-promoting capabilities for dynamic infected burn wound healing.

**Scheme 1:**
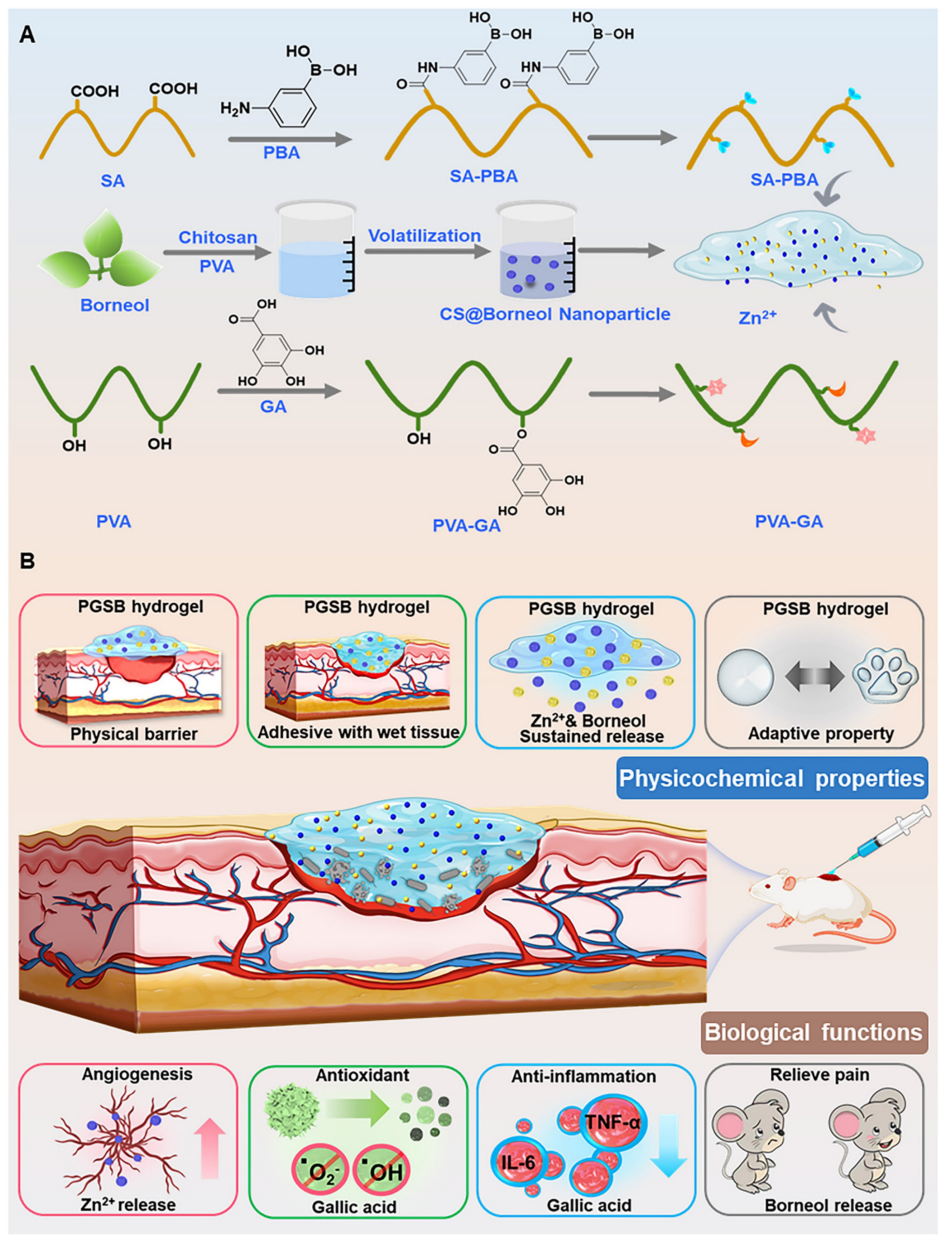
(A) Preparation of hydrogels and their components. (B) Illustration of the physicochemical properties (physical protection, tissue adhesion, Zn^2+^ and borneol release, and shape adaptability) and biological functions (angiogenesis-promoting, antioxidant, anti-inflammatory, and pain-reliving effects) of the PGSB enabling the effective treatment of* S. aureus* infected burn wounds.

Herein, PVA grafted with GA (PVA-GA), SA grafted with 3-aminobenzeneboronic acid (PBA; SA-PBA), Zn^2+^ ions, and CS@BN nanoparticles are combined to afford a hydrogel (PGSB) physicochemically crosslinked via (i) hydrogen bonding, (ii) ion/coordination interactions of Zn^2+^ with SA-PBA/PVA-GA, and (iii) dynamic boronic ester bonds between PVA-GA and SA-PBA (Scheme 1). PGSB exhibits enhanced mechanical properties and an outstanding self-healing, injectability, shape adaptability, and adhesive strength. The integration of important biological functions (pain-relieving, antibacterial, anti-inflammatory, vascularization-promoting, and anti-oxidant activities) in a single hydrogel platform, makes it suitable for the treatment of dynamic, irregular, and infected burn wounds. The wound healing ability of PGSB is demonstrated in an *in vivo S. aureus* infected murine burn wound model. Compared with a commercial burn cream, PGSB markedly accelerates infected burn wound healing, provides pain relief (as revealed by the decreased mouse grimace scale (MGS) scores and paw lifting and licking times), and shortens the wound healing time to 14 days.

## Methods

### Syntheses of PVA-GA and SA-PBA

PVA-GA was synthesized as follows: PVA (0.25 g) was dissolved in deionized (DI) water (15 mL), and the solution was treated with GA powder (0.48 g) and then dropwise supplemented with a solution of DMAP (0.35 g) in DI water (3 mL) under N_2_. After 48 h, the dark brown solution was collected and dialyzed (molecular weight cut-off =14000 Da) for three days. The purified solution was freeze-dried to obtain PVA-GA (brown), which was stored in a vacuum dryer for further use.

SA-PBA was synthesized according to the following steps: SA (0.5 g) was dissolved in MES buffer with pH 6 (80 mL, 50 mM). The solution was treated with DMTMM (0.7 g) to activate the carboxyl groups of SA and then supplemented with PBA (0.34 g). After 48 h at room temperature, the solution was dialyzed (molecular weight cut-off =14000 Da) and lyophilized to obtain SA-PBA.

### Synthesis of CS@BN nanoparticles

The CS@BN nanoparticles were prepared using an emulsion method 44, 45. CS (0.05 g) was dissolved in an aqueous PVA solution (5 mL, 5 wt%) containing acetic acid, and the resulting mixture was treated with a solution of borneol in CH_2_Cl_2_ (50 μL, 100 mg mL^-1^) to form an emulsion. CH_2_Cl_2_ was evaporated under vigorous stirring to afford a light-yellow transparent solution of the CS@BN nanoparticles (1 mg mL^-1^).

### Preparation of hydrogels

The borneol-free hydrogel (PGS) was prepared according to the following steps: Aqueous solutions of PVA-GA (20 wt%), SA-PBA (0.75 wt%), and ZnSO_4_ (0.1 M) were mixed in a 20:50:5 volume ratio. The PGS formed several seconds after mixing exhibited excellent self-healing due to the dynamic boronic ester bonds between PVA-GA and SA-PBA, interactions of Zn^2+^ with PVA-GA (Zn^2+^ coordination by the polyphenol structure) and SA-PBA (attraction between Zn^2+^ and carboxylate ions), and hydrogen bonding.

PGSB was prepared in the same way, with PVA-GA dissolved in the CS@BN nanoparticle solution instead of water.

### Characterizations

Chemical structures were probed by Fourier transform infrared spectroscopy (Thermo Scientific Instrument, Nicolet 6700) and ^1^H nuclear magnetic resonance (NMR) spectroscopy. The morphologies of PGSB and the CS@BN nanoparticles were characterized by scanning electron microscopy (SEM; Zeiss, Gemini360) and transmission electron microscopy (JEOL, JEM-2100F), respectively. Rheological properties, self-healing, and injectability were investigated using a TA rheometer (DHR-2). The swelling behaviors and degradability of PGS and PGSB were examined by weighing the samples after different times of immersion into phosphate-buffered saline (PBS; pH 7.4 and 5.8). Adhesive properties were probed by examining adhesion to different substrates. Further hydrogel characterization details are provided in the Supporting Material.

### *In vitro* biocompatibility evaluation

*In vitro* biocompatibility was examined using cyto- and hemocompatibility tests. The cytocompatibilities of PGS and PGSB (100 μL) were tested using a live/dead assay and CCK8 cell viability kit with 200 μL of culture media in a 48-well plate for the designated culture time. The hemocompatibilities of PGS and PGSB were expressed as the hemolysis fraction of mouse blood cells after 1 h of coincubation.

### *In vitro* antioxidant performance evaluation

The 1,1-diphenyl-2-picrylhydrazyl (DPPH) radical scavenging activity assay was used to evaluate the antioxidant performances of PGS and PGSB. The homogenized hydrogel (100 µL) was thoroughly mixed with an methanolic DPPH solution (200 µL, 100 µM), and the mixture was incubated at 37 °C for 30 min in the absence of light. Ascorbic acid (100 μM) was used as a positive control.

### *In vitro* cell migration and angiogenesis evaluation

Cell migration was investigated using a scratch experiment with 100 μL of PGS or PGSB and 400 μL of culture medium in a 24-well plate. Proangiogenic activity was examined by characterizing the expression of the platelet endothelial cell adhesion molecule (CD31) using reverse transcription polymerase chain reaction (RT-PCR).

### *In vitro* anti-inflammatory activity evaluation

Cells were subjected to lipopolysaccharide-induced macrophage polarization for one day and then cultured with the hydrogel of choice for two days. The macrophage RNA was collected and reverse-transcribed to obtain the related gene expression (tumor necrosis factor α (TNF-α) and interleukin 6 (IL-6)) indicating inflammation.

### Antibacterial property evaluation

*S. aureus* (Gram-positive) and ampicillin-resistant *E. coli* (Gram-negative) were used to investigate the antibacterial activities of PGS and PGSB. The bacterial suspension (100 μL, optical density (OD) = 1) was diluted to the desired concentration and dropped into PGS or PGSB (200 μL) placed in a 24-well plate. The mixture was incubated in an automated shaker at 37 °C for 2 h and then diluted with PBS (900 μL). A 100 μL aliquot of the diluted bacterial suspension was collected and cultivated on the plate for 24 h. The plate was photographed, and the bacterial killing ratio (%) was calculated as 100% × (colony count of control - colony count of hydrogel)/colony count of control.

### Infected burn wound healing study

Animal experiments were conducted in accordance with the guidelines of the Animal Research Committee of Xi'an Jiaotong University (No. XJTUAE2024-1940). The *in vivo* wound healing abilities of PGS and PGSB were studied using a *S. aureus* infected burn wound model established using female Kunming mice. A steel rod was heated to 100 °C and used to burn the smooth skin of the mice for 10 s to form a circular scalded area. A suspension of *S. aureus* (50 μL, 10^7^ CFU mL^-1^) was subcutaneously injected into the burned skin, and the infected wound was treated with the material of choice (blank control, commercial burn cream, PGS, or PGSB). After 3, 7, and 14 days, the wound area was photographed and analyzed using the Image J software.

### Pain-relieving effect evaluation

The MGS evaluation was conducted according to a previous study 46 to characterize the pain-relieving ability of PGSB. After *S. aureus* infection, mouse grimaces were recorded based on five independent facial action units (cheek bulge, nose bulge, orbital tightening, whisker position, and ear position), and each action unit was scored on a three-point scale (0, 1, or 2). The MGS scores were obtained by summing the scores of the five independent facial action units.

### Histological and immunofluorescence staining evaluation

Wound samples were collected at 3, 7, and 14 days after treatment. Hematoxylin and eosin (H&E) staining was used to investigate capillary regeneration and inflammation. TNF-α and CD31 immunofluorescence staining was used for inflammation and angiogenesis testing, respectively.

### Statistical analysis

Experimental results were expressed as means ± standard deviations, and differences were considered significant at *p* < 0.05. Statistical analysis was performed according to the Student's *t*-test.

## Results and Discussion

### Design and physicochemical characteristics of the CS@BN nanoparticles and hydrogels

**Figure 1:**
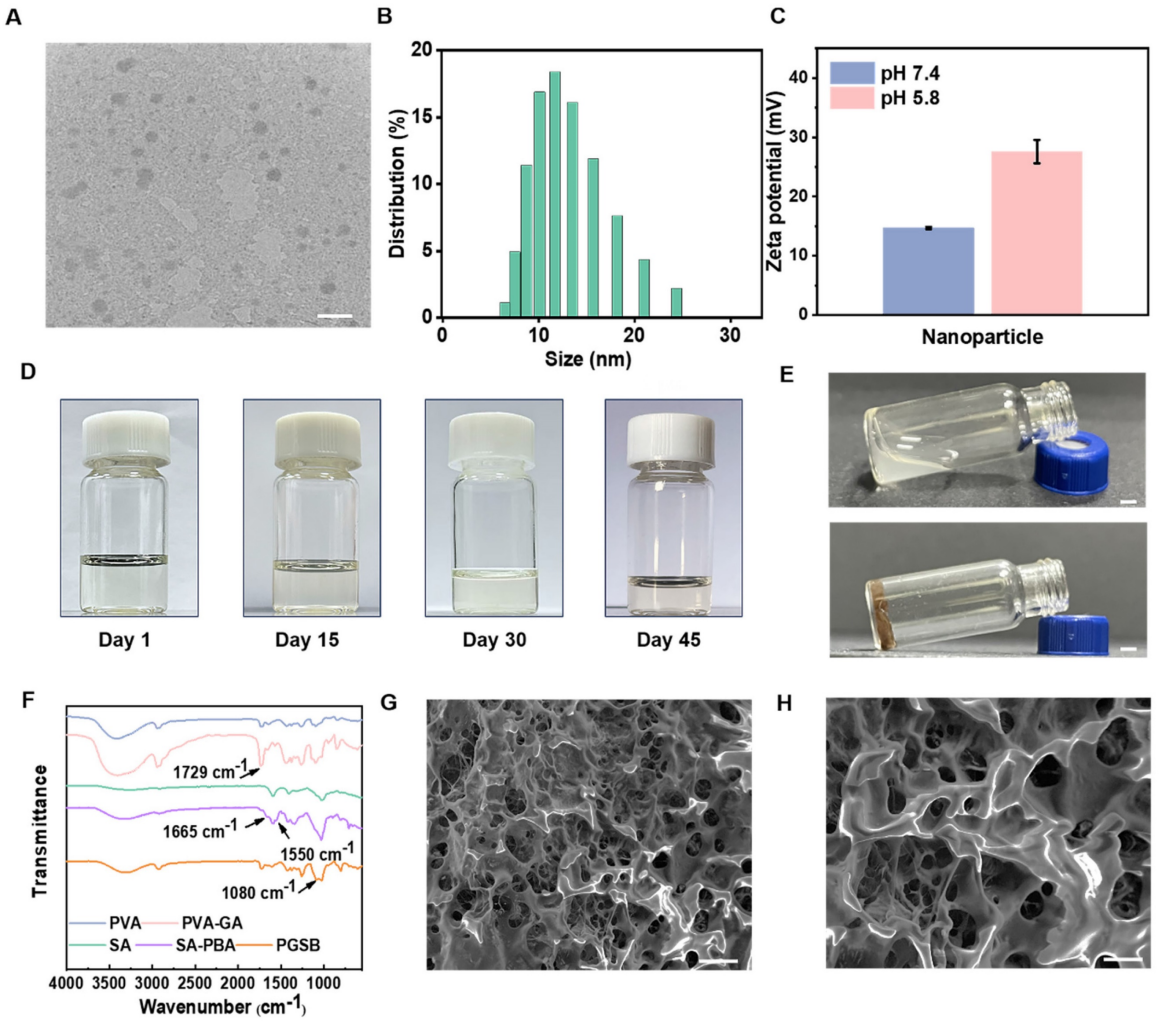
Characterization of the CS@BN nanoparticles and PGSB. (A) TEM image (scale bar = 50 nm), (B) size distribution, (C) zeta potential diagram, and (D) stability diagram of the CS@BN nanoparticles. (E) Illustration of PGSB formation. The flowing liquid in the upper glass bottle is SA-PBA solution, and the fixed sample in the lower glass bottle is PGSB. Scale bar = 0.25 cm. (F) Fourier transform infrared spectra of PGSB and its matrix components. (G, H) SEM images of PGSB (scale bar = 100 μm in (G) and 50 μm in (H)).

The CS@BN nanoparticles displayed a uniform spherical morphology and desirable diameters of 10‒20 nm (Figures 1A-B). The stability of these nanoparticles over the course of two weeks was tested using dynamic light scattering (DLS) measurements (Figure S1). The size distributions of the nanoparticles on days 1, 7, and 14 were similar, indicating a high stability. CS is a pH-responsive polymer soluble in acidic media. Zeta potential measurements (Figure 1C) indicated that the CS@BN nanoparticles were more positively charged in acidic environments than in alkaline ones, which was ascribed to the accumulation of positive charge due to the ionization and dissolution of CS in acidic media. Bacterial infections can generate acidic metabolites, including lactic and carbonic acids, resulting in acidic wounds 5. The extra borneol could thereby be smartly released in infected wounds due to dissolution of the CS coating to facilitate their treatment. The stability of the CS@BN nanoparticles was confirmed by the absence of aggregation/sedimentation/turbidity in the corresponding solution over 45 days of storage (Figure 1D).

The triply physicochemically crosslinked hydrogel (PGSB) was designed by considering the features of dynamic infected burn wounds. PVA and SA were selected as the major matrices because of their excellent biocompatibility and degradability. The bioactive GA and PBA were separately grafted onto PVA and SA to form PVA-GA and SA-PBA, respectively, which enabled crosslinking through the formation of dynamic boronic ester and hydrogen bonds. Zn^2+^, which exhibits antibacterial and pro-vascularization properties, was introduced to form further physical crosslinks through PVA-GA/Zn^2+^ and SA-PBA/Zn^2+^ coordination. Consequently, the triple physicochemically crosslinked PGSB hydrogel was constructed. The dynamic boronic ester, metal coordination, and hydrogen bonds enhanced not only the mechanical strength of PGSB but also its bioactivity, while the CS@BN nanoparticles provided cooling, pain-easing, and anti-inflammatory properties.

The hydrogel formation mechanism is illustrated in Figure 1E. The peaks at 7.2-7.8 ppm in the ^1^H NMR spectrum of SA-PBA (Figure S2) were ascribed to benzene ring attached hydrogens and confirmed the grafting of PBA onto SA, while the peaks at 6.9, 8.8, and 9.2 ppm in the spectrum of PVA-GA indicated that GA was successfully grafted onto PVA. The emergence of a new peak at 1729 cm^-1^ in the Fourier transform infrared spectrum of PVA-GA (Figure 1F) confirmed the formation of ester bonds, while the newly emerged peaks at 1665 and 1550 cm^-1^ in the spectrum of SA-PBA were assigned to amide bonds and confirmed the reaction between the amino and carboxyl groups. The peak at 1080 cm^-1^ observed for PGSB indicated boronic ester formation due to the crosslinking reaction between PVA-GA and SA-PBA. SEM imaging (Figures 1G-H) revealed that PGSB had a typical porous structure that enabled wound exudate absorption and nutrient/oxygen supply and thereby facilitating wound healing. The combined results confirmed the preparation and desired structure of PGSB.

### Rheology, self-healing, injectability, swelling/degradation, drug release, adhesive, and shape-adaptable properties of the hydrogels

**Figure 2:**
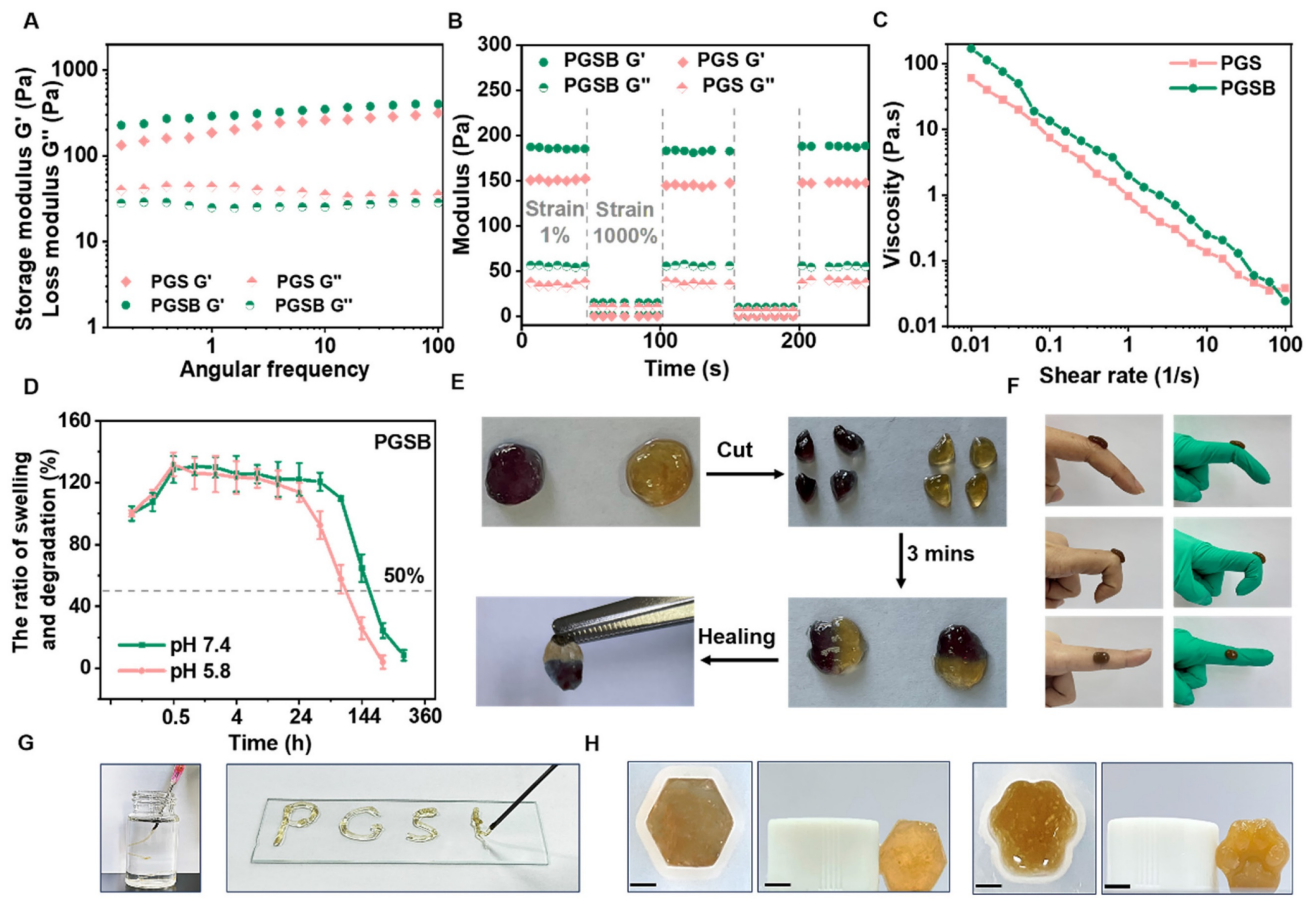
Multifunctional properties of the hydrogels. (A) Storage (*G*′) and loss (*G*′′) moduli of the PGS and PGSB at 37 °C. (B) Evolution of the *G*′ and *G*′′ of PGS and PGSB during three strain cycles from 1% to 1000%. (C) Effects of shear rate (0.01 to 100 s^-1^) on the viscosities of PGS and PGSB. (D) Swelling and degradation of PGSB at pH 7.4 and 5.8. (E) Images showing the self-healing of PGSB at room temperature. (F) Adhesion of PGSB to skin and rubber determined at angles of 45°, 90°, and 180°. (G) Images illustrating the injectability of PGSB. (H) Shape adaptability of PGSB. Scale bar = 0.25 cm.

For both PGS and PGSB, the storage modulus exceeded the loss modulus, which indicated a stable gel state (Figure 2A). Owing to their structural similarity, PGS and PGSB demonstrated similar storage moduli (150-200 Pa). The hydrogel obtained without Zn^2+^ could not maintain its morphology and collapsed (Figure S3A), showing a storage modulus (~45 Pa) markedly lower than those of PGS and PGSB (Figure S3B). The self-healing behavior of PGSB was examined using the step-strain sweep mode (Figure 2B). PGSB adopted a normal gel state (*G*′ > *G*′′) at a low strain (1%) and a sol state (*G*′ < *G*′′) at a high strain (1000%). During three repeated cycles, the storage modulus of PGSB recovered to its original value, which indicated an excellent self-healing property. When circular hydrogel samples were cut into four pieces (Figure 2E), an autonomous restoration of the original morphology was observed when these pieces were brought into contact with each other. This high self-healing was ascribed to the dynamic physicochemical bonds in PVA-GA/SA-PBA (including boronic ester bonds), PVA-GA/Zn^2+^ (coordination of Zn^2+^ by phenolic hydroxyl), SA-PBA/Zn^2+^ (coulombic interactions between Zn^2+^ and carboxylate anions), and multiple hydrogen bonds. The injectability of PGSB was assessed by determining its shear-thinning behavior (Figure 2C). The viscosity of PGS and PGSB decreased with the increasing shear rate, which demonstrated desirable shear-thinning properties. Moreover, PGSB could be smoothly injected from a 5 mL syringe without fracturing (Figure 2G) to continuously form alphabetical characters, e.g., “PGSB”. These characteristics are expected to facilitate the application of PGSB in dynamic wound scenarios.

The swelling and degradation behaviors of PGSB were pH-dependent (Figure 2D). Specifically, the times of swelling equilibrium establishment at pH 7.4 and 5.8 were ~1 and 0.5 h, respectively, and the corresponding ultimate swelling ratios were similar (131% and 132%, respectively). This behavior was expected to benefit wound exudate absorption. PGSB underwent slow and stable degradation over the first three days and rapid degradation during the remaining nine days, showing an ultimate weight loss of 92% at pH 7.4. At pH 5.8, PGSB showed a weight loss of 8% in the first two days and 74% in the following four days. PGS featured a similar degradation behavior (Figure S4) because its crosslinking structure was similar to that of PGSB. However, some hydrogels crosslinked by single interactions undergo rapid degradation with over 50% weight loss within two days, especially in acidic environments 5. In the first two days, PGSB exhibited a notably slower degradation than some hydrogels crosslinked by single interactions, i.e., the degradation rate of the former better matched the wound healing rate.

The pH-responsive degradation behavior of PGSB due to crosslinking via boronate ester and coordination bonds inspired us to investigate the pH-dependent release of Zn^2+^ and the borneol (Figure S5). The release rates of these species were higher in a mildly acidic environment owing to the impair of boronate ester and coordination bonds. The release rate of the borneol after 24 h was 41.2% at pH 5.8 but only 10.1% at pH 7.4. When the release time was extended to three days, these values increased to 70.0% and 30.1%, respectively, while values of 84.1% (pH 5.8) and 45.9% (pH 7.4) were observed after four days. Similarly, the Zn^2+^ release rates at pH 5.8/7.4 equaled 57.0%/21.3% after one day, increased to 76.9%/52.7% after seven days, and minimally increased further after 14 days. Bacterial wound infections generally create acidic environments; thus, the promotional effects of such environments on the release of the borneol and Zn^2+^ ions were expected to facilitate the healing of infected burn wounds.

Conventional non-adhesive hydrogel dressings need to be fixed at wound sites using fixation tools and therefore have unstable connections to dynamic wounds. Conversely, adhesive hydrogel dressings can completely and seamlessly adhere to dynamic wounds, preventing their infection 47. GA has been reported to improve the strength of hydrogel adhesion to skin and promote dynamic movement in wound applications 48. Hence, we examined the ability of PGSB to adhere to different substrates (Figures 2F, S6, and S7). The amount of PGSB required to adhere to and lift tissue substrates (including kidney, liver, heart, skin, and lung tissues) was as low as 100 μL, while that required for plastic, rubber, steel, and glass was only 300 μL. In addition, a 50 g smooth metal weight was lifted using 300 μL of PGSB, which illustrated the excellent adhesion properties of this hydrogel. The lap shear test used to probe the adhesion of PGSB to pig skin (Figure S8) showed that PGSB exhibited an adhesive strength of 19.6 kPa, which was comparable with that reported in recent studies on adhesive hydrogels for wound healing 49, 50.

Owing to the irregular shape of typical burn wounds, we further investigated the shape adaptability of PGSB, i.e., its ability to adapt to various wound morphologies, by transferring PGSB prepared in a hexagonal mold (Figure 2H) to a cat claw-shaped mold. The transferred hydrogel adopted the shape of the new mold and thus exhibited an excellent shape adaptability. Shape adaptability was further quantified by characterizing the storage modulus retention of PGSB after shaping. The values obtained for hexagonal (98.6%) and cat-claw (97.5%) samples indicated an outstanding shape adaptability (Figure S9). These results showed that PGSB was well suited for the treatment of irregularly shaped wounds.

### *In vitro* cytocompatibility and hemocompatibility

**Figure 3:**
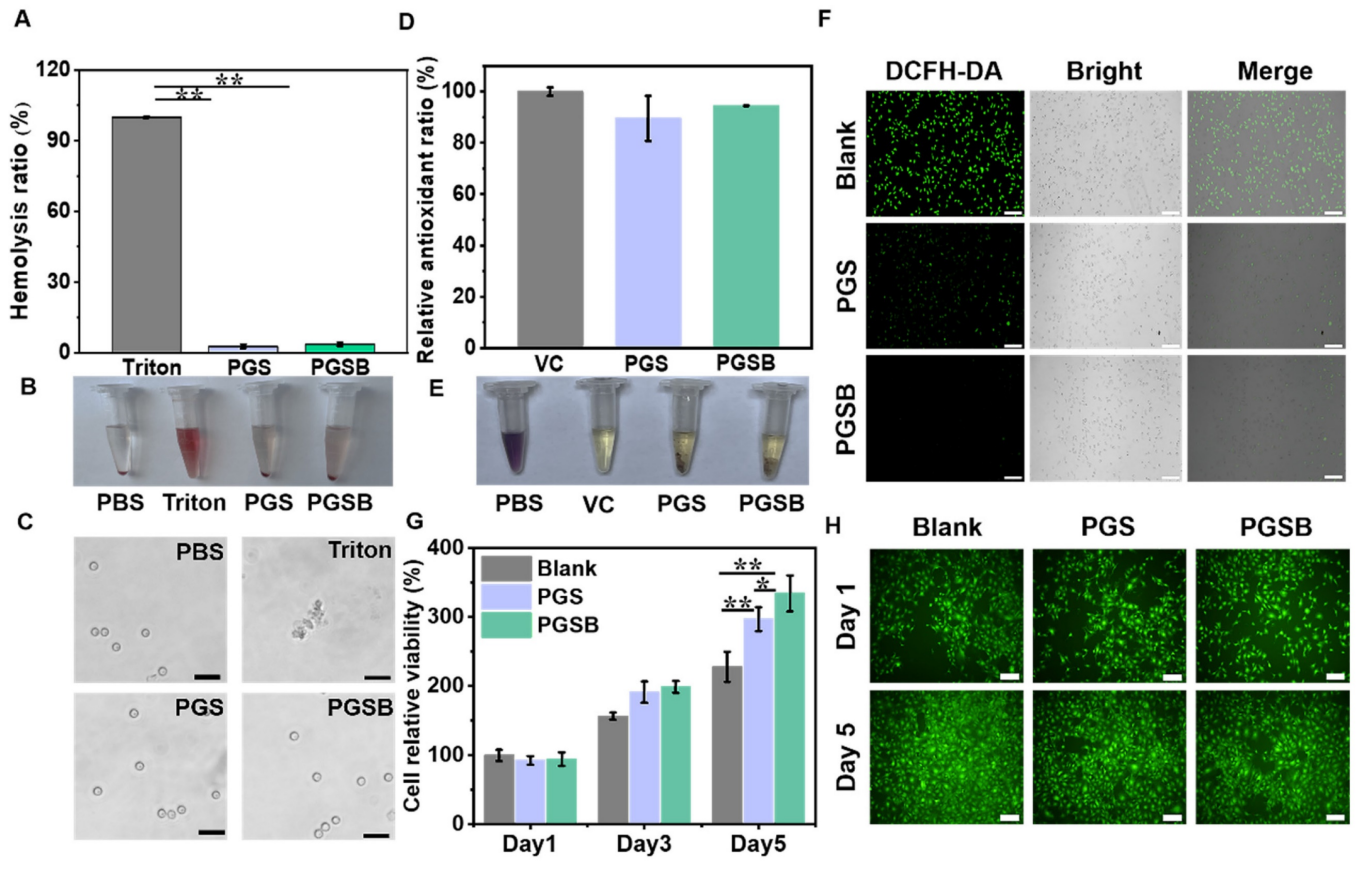
Hydrogel biocompatibility and antioxidant properties. (A) Hemolysis ratio of red blood cells after incubation with the hydrogels and Triton X-100 (complete hemolysis control), “Triton” represents Triton X-100. (B) Macroscopic photographs of red blood cell suspensions after centrifugation, “Triton” represents Triton X-100. (C) Microscopic photographs of red blood cells incubated with the hydrogels (scale bar = 20 μm). (D) Antioxidant activities of the hydrogels (control = ascorbic acid (VC)). (E) Photographs of methanolic DPPH solutions after 30 min of incubation with the desired hydrogel or VC. (F) Fluorescence images of the L929 cells cocultured with the hydrogel, with green fluorescence indicating intracellular reactive oxygen species (scale bar = 100 μm). (G) Viability of the L929 cells after five days of coculturing with the hydrogels. (H) Live/dead staining images of the L929 cells on days 1 and 5 of coculturing with the hydrogels (scale bar = 50 μm; green fluorescence indicates live cells).* n* = 4, *p < 0.05, **p < 0.01.

The results of cytocompatibility and hemocompatibility testing are shown in Figure 3. Compared with the positive control group treated with Triton X-100, the groups treated with PGS and PGSB displayed low hemolysis ratios of 2.7% and 3.6%, respectively, which indicated the good hemocompatibility of these hydrogels (Figure 3A). A bright red color was observed in the positive control group due to 100% cell rupture, whereas a faint red color was observed in the PBS, PGS, and PGSB groups (Figure 3B). Hemocompatibility was further examined by inspecting blood cells under a microscope (Figure 3C). The blood cells displayed a normal round shape in the PBS, PGS, and PGSB groups but were completely ruptured in the positive control group. The cytocompatibility of PGS and PGSB was studied using a cell proliferation test and the live/dead staining of L929 cells. The proliferation of the L929 cells cocultivated with PGS and PGSB was investigated by testing cell viability (Figure 3G). On each day (days 1, 3, and 5), the viability of the cells in the blank group seeded on a 48-well plate was similar to that of the cells in the PGS and PGSB groups. With an increase in the cultivation time, the cell viability increased compared with that on day 1 (~3.2-fold on day 5 for the PGS group and ~3.5-fold on day 5 for the PGSB group). In addition, the morphology of the L929 cells was examined after cocultivation with PGS and PGSB for one and five days. Most cells showed a normal, live, spindle-like morphology (green cells) during the cocultivation period (Figure 3H). We further tested the viability of cells cocultivated with different hydrogel amounts (Figure S10), revealing that a high viability comparable with that of the blank group was observed in all cases. The cytocompatibility and hemocompatibility testing results indicated the outstanding biocompatibility of PGS and PGSB.

### *In vitro* antioxidant activity

Typically, hydrogel wound dressings with antioxidant properties have numerous advantages, including the ability to ameliorate the wound microenvironment by regulating reactive oxygen species (ROS) and thereby accelerate wound healing 17, 51. Similar to dopamine, GA can enhance the antioxidant activity of hydrogels 52, 53. DPPH elimination ratios of 100%, 89.5%, and 94.5% were observed for the positive control (ascorbic acid), PGS, and PGSB groups, respectively, indicating that the incorporation of the CS@BN nanoparticles enhanced antioxidant properties (Figure 3D). Macroscopic observations showed that the dark purple DPPH solution turned pale yellow upon exposure to ascorbic acid, PGS, and PGSB (Figure 3E). Intracellular ROS levels were studied by fluorescence imaging to further examine the antioxidant properties of PGS and PGSB (Figure 3F). The blank group showed green fluorescence indicating high level of intracellular ROS, whereas the PGS and PGSB groups displayed negligible fluorescence intensities. Overall, PGS and PGSB had high antioxidant activities benefiting wound healing applications.

### *In vitro* cell migration-promoting, provascularization, and anti-inflammation effects

**Figure 4:**
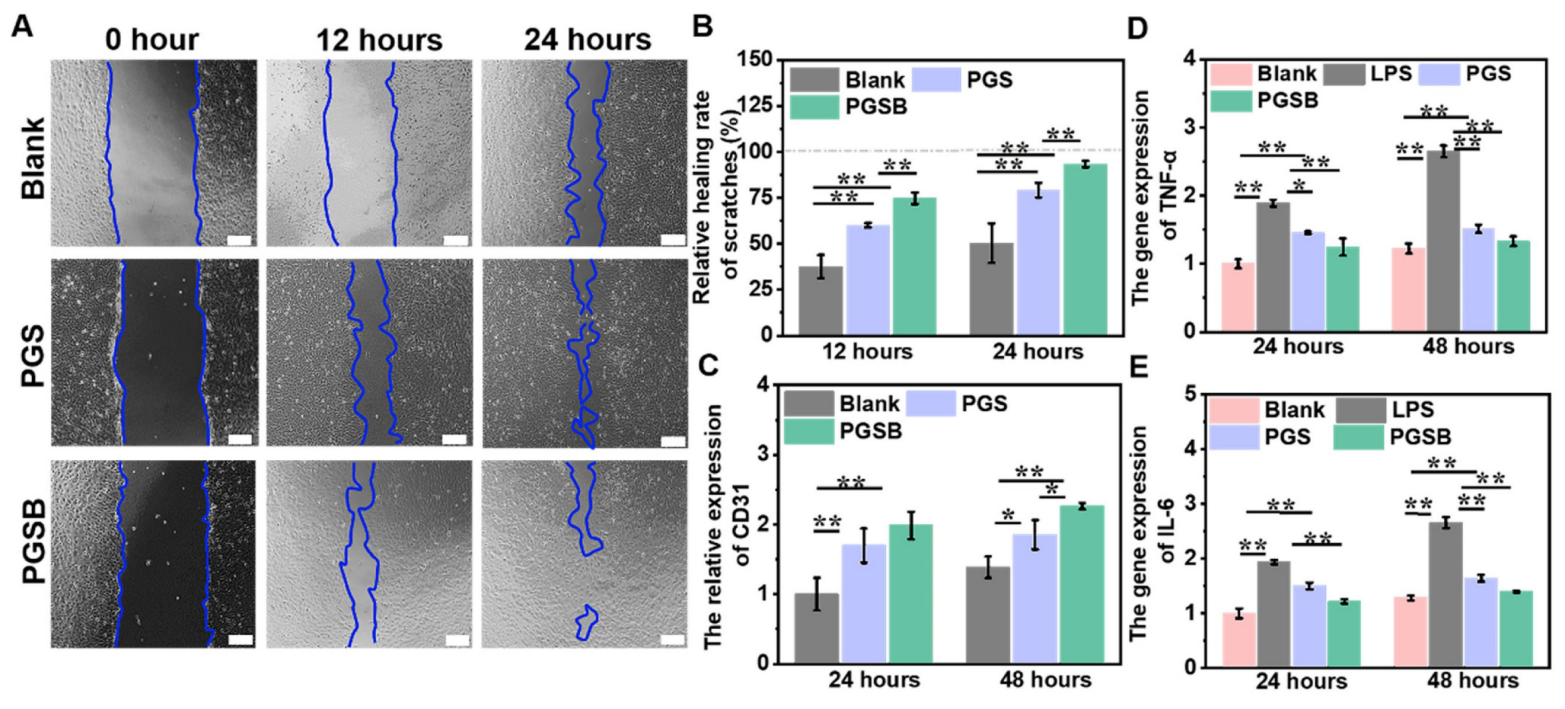
*In vitro* cell migration-promoting, proangiogenic, and anti-inflammatory properties of the hydrogels. (A) Proliferation and migration images of HUVECs cocultured with the hydrogels (scale bar = 200 μm). (B) Healing rates obtained in scratch experiments. (C) Platelet endothelial cell adhesion molecule (CD31; angiogenesis marker) gene expression. (D) Tumor necrosis factor α (TNF-α; inflammatory marker) gene expression. (E) Interleukin 6 (IL-6; inflammatory marker) gene expression.* n* = 4, *p < 0.05, **p < 0.01.

Cell migration is defined as the directed movement of cells triggered by chemical or mechanical signals and plays a pivotal role in tissue renewal and wound repair, as wound repair requires the organized movement of cells to specific destinations 54. The abilities of PGS and PGSB to promote cell migration were studied using cell scratch experiments, with wound areas at 0, 12, and 24 h determined using a microscope (Figure 4A). The PGSB group demonstrated a wound area smaller than those in the control and PGS groups. In addition, the wound area in the control group significantly exceeded those in the PGS and PGSB groups at 12 and 24 h. Specifically the wound area in the control group after 24 h was 2.4- and 7.5-fold larger than those in the PGS and PGSB groups, respectively, which demonstrated that PGS and PGSB effectively promoted cell migration. The scratch healing rate in the PGSB group reached 93.4% after 24 h (Figure 4B). Figure 4C presents the results of provascularization property testing, revealing that CD31 expression in the PGS and PGSB groups significantly exceeded that in the blank group at 24 and 48 h, with the highest values observed in the PGSB group. Specifically, the CD31 expression in the PGSB group at 24 and 48 h was ~2.0- and ~1.6-fold higher than that in the blank group, respectively, demonstrating the excellent ability of PGSB to promote cell vascularization.

Anti-inflammation activity is important for promoting wound healing, as inflammation is the second process of wound healing and can lead to a variety of symptoms, including redness, pain, swelling, warmth, and even function loss, which may delay healing. Consequently, numerous anti-inflammatory hydrogel dressings have been developed to accelerate wound healing 40, 55. GA and borneol have been reported to exhibit anti-inflammatory activity 48, 56. Figures 4D-E present the anti-inflammatory effects of PGS and PGSB determined by measuring the gene expression of inflammatory cytokines, including TNF-α and IL-6, by RT-PCR. After treatment for 24 and 48 h, the gene expression of both IL-6 and TNF-α in a single lipopolysaccharide-inducted group significantly exceeded that in the blank group (*p* < 0.01). In the PGS and PGSB groups, IL-6 and TNF-α gene expression was significantly downregulated compared with that in the single lipopolysaccharide-inducted group. PGSB exhibited the best anti-inflammatory ability, as it suppressed inflammatory gene expression, which was ascribed to the presence of bioactive components (GA and CS@BN nanoparticles).

### Antibacterial activity

As mentioned earlier, burn wounds are susceptible to bacterial (e.g., *S. aureus* and* P. aeruginosa*) infections, which cause complications (e.g., acidic environments and inflammation) resulting in delayed wound healing 57. Antibacterial activity is another essential characteristic of hydrogel dressings used to treat infected wounds. Zn^2+^, GA, and CS have desirable antibacterial activity 48, 58.

**Figure 5:**
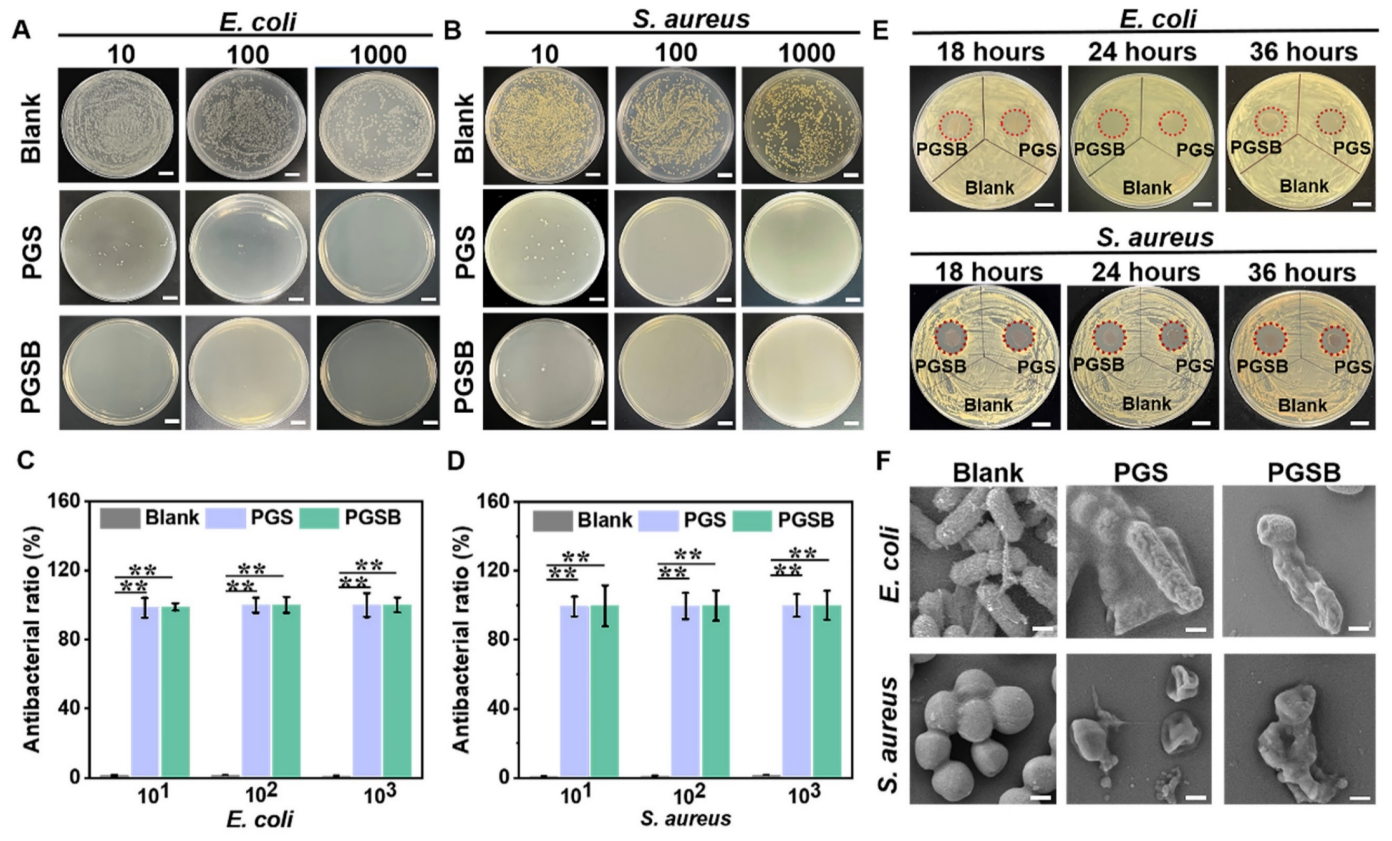
Antibacterial activities of PGS and PGSB. (A) Suspensions of *E. coli* with different dilution ratios cocultured with PGS and PGSB for 2 h survived on agar plates. Scale bar = 1 cm. (B) Suspensions of *S. aureus* with different dilution ratios cocultured with PGS and PGSB for 2 h survived on agar plates. Scale bar = 1 cm. (C) Activities of PGS and PGSB against *E. coli*. (D) Activities of PGS and PGSB against *S. aureus*. (E) Inhibition zones of PGS and PGSB after 36 h incubation with *E. coli* and* S. aureus* on agar plates. Scale bar = 1 cm. (F) SEM images of *E. coli* and* S. aureus* after 2 h incubation with PGS and PGSB (scale bar = 200 nm).* n* = 4, *p < 0.05, **p < 0.01.

The antibacterial activities of PGS and PGSB were tested using *S. aureus* and* E. coli*. Bacterial suspensions of different concentrations were cocultured with PGS or PGSB for 2 h (Figures 5A-D) and then cultivated on a plate with a solid medium for 24 h (Figures 5A and C). No bacterial colonies were observed in the PGS or PGSB groups when the dilution ratio of the bacterial suspensions was 100 or 1000. Conversely, full plate coverage with bacterial colonies was observed in the control group. When the dilution ratio was set to 10, the antibacterial activity in the PGSB group exceeded that in the PGS group. Almost 99% of *S. aureus* and* E. coli* was eliminated in the PGS and PGSB groups at different dilution ratios (Figures 5B and D). These data demonstrated the high bactericidal activities of PGS and PGSB. In addition, we tested the antibacterial activities of PGSB crosslinked using different concentrations of Zn^2+^ (Figure S11). The hydrogel obtained in the absence of Zn^2+^ demonstrated a minimal activity against *S. aureus* and *E. coli*, possibly because the GA and CS@BN nanoparticle loadings of PGSB were insufficient for effective eradication. When the Zn^2+^ concentration was increased to 0.05 M, the activity against *S. aureus* and *E. coli* increased, although some bacteria survived. Conversely, elimination was almost complete at Zn^2+^ concentrations of 0.1 M (Figures 5A-D) and 0.2 M. Given that high Zn^2+^ concentrations would lead to a higher crosslinking density and thus result in a strong but brittle hydrogel, we selected a Zn^2+^ concentration of 0.1 M for further study. The excellent antibacterial properties of PGS and PGSB were further verified in liquid media (Figure S12). The OD_600_ values of both *S. aureus* and *E. coli* in the PGS and PGSB groups remained at a very low level when the cocultivation time was increased to 36 h. Conversely, the OD_600_ value for the blank group sharply increased with the increasing cultivation time. The results of testing in liquid media further confirmed the excellent antibacterial properties of PGS and PGSB.

The antibacterial activities of PGS and PGSB were further quantified by measuring the diameters of the corresponding inhibition zones at predetermined time points (Figures 5E and S13). In the case of *E. coli*, inhibition zones with diameters of 15.9 mm (18 h), 18.0 mm (24 h), and 18.0 mm (36 h) for PGS group, and 17.8 mm (18 h), 21.6 mm (24 h), and 21.0 mm (36 h) were observed in the PGSB group. The diameter of the inhibition zone was not reduced in the PGS and PGSB groups when the incubation time was increased, and remarkable bactericidal activity against* E. coli* was maintained for 36 h. Similar results were obtained for *S. aureus* (Figures 5E and S13), with the inhibition zone diameters after 18 h equaling 17.0 mm (PGS) and 18.7 mm (PGSB), respectively, and thus demonstrating a high sterilization ability. After 36 h of incubation, the inhibition zone diameters in the PGS and PGSB groups did not significantly decrease, which confirmed the excellent antibacterial activities of these hydrogels against *S. aureus.* The antibacterial properties of PGS and PGSB were further verified by bacterial morphology observation*.* In the blank control group, *E. coli* and* S. aureus* showed normal morphologies (*E. coli*: rod-like, *S. aureus*: spherical) without shrinkage or collapse (Figure 5F). After PGS or PGSB treatment, both *E. coli* and* S. aureus* were killed, as revealed by their wrinkled morphologies. Thus, PGS and PGSB exhibited high antibacterial activities against Gram-positive and -negative bacteria in both liquid and solid environments, holding promise for the healing of infected burn wounds. The antibacterial activity of PGSB exceeded that of PGS because of the presence of the CS@BN nanoparticles in the former hydrogel.

### *In vivo* effects of the hydrogels on the healing of *S. aureus* infected deep burn wounds

**Figure 6:**
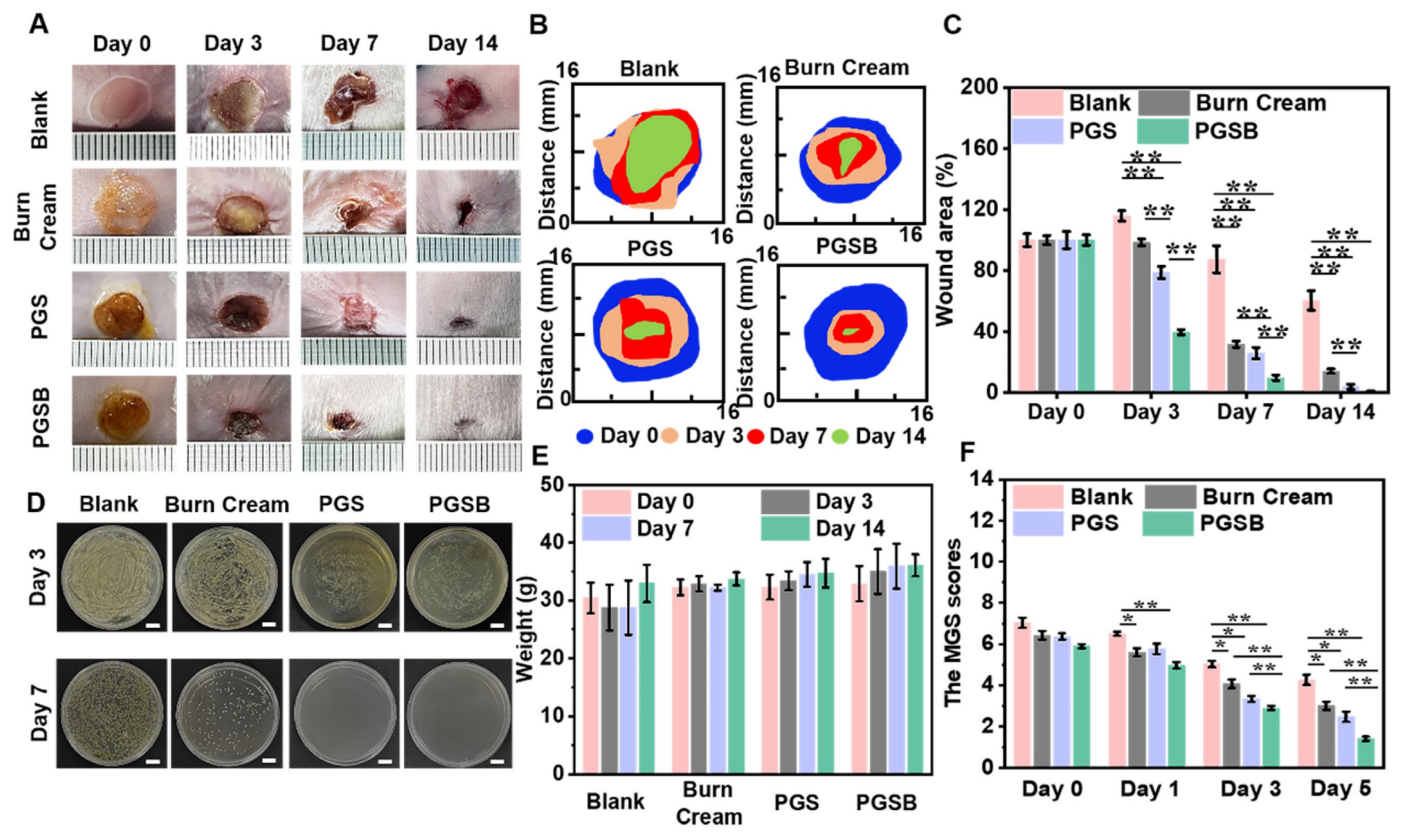
*In vivo* infected deep burn wound healing efficacies of the hydrogels. (A) Representative images of infected burn wounds in different groups after 0, 3, 7, and 14 days of treatment. (B) Diagram of the dynamic wound healing process in different groups after 0, 3, 7, and 14 days of treatment. (C) Wound area closure ratios in different groups. (D) Antibacterial activity of PGSB in the healing of infected burn wounds. Scale bar = 1 cm. (E) Mouse weights in different groups after 0, 3, 7, and 14 days of treatment. (F) Mouse grimace scale scores of different groups after 0, 1, 3, and 5 days of treatment. *n* = 4, *p < 0.05, **p < 0.01.

Given that burn wounds are vulnerable to infections by bacteria, such as *S. aureus*, we examined the *in vivo* abilities of PGS and PGSB to promote the healing of *S. aureus-*infected deep burn wounds. On day 3, the wound area increased by 15.9% in the blank group, whereas a 1.5% contraction was observed in the burn cream group because of the severe wound infection, while the wounds in the PGS and PGSB groups contracted by 21.1% and 60.4%, respectively (Figures 6A-C and S14). Thus, significant differences between the control and hydrogel groups were observed. With the increasing treatment time, the wound area decreased in all groups. After seven days of treatment, the wounds were minimally infected in the blank and burn cream groups because of the limited antibacterial activity of the cream, with the respective wound closure ratios equaling 12.6% and 68.2%. For comparison, higher and significantly different wound closure ratios of 74.0% and 90.2% were observed in the PGS and PGSB groups, respectively. After 14 days of treatment, wound closure ratios of 99.1%, 96.1%, 39.6%, and 85.9% were observed in the PGSB, PGS, blank, and burn cream groups, respectively. The bacterial infection of the wounds was also investigated on days 3 and 7 (Figures 6D and S15). Severe infections were observed on day 3 in the blank and burn cream groups, whereas mild infections were observed in the PGS and PGSB groups. On day 7, severe infection was observed in the blank group, and a moderate infection was observed in the burn cream group, whereas no infection was observed in the PGS and PGSB groups, which indicated the high *in vivo* antibacterial activities of these hydrogels. Furthermore, PGSB could be used to detect electrocardiogram and surface electromyography signals (Figure S16), thus holding promise for wound state monitoring through electrical signal detection 59, 60. These results indicated that PGSB was suitable for treating infected deep burn wounds. The therapeutic effects of PGS and PGSB surpassed those of the commercial burn cream. PGSB was shown to accelerate infected burn wound healing and shorten the healing period.

### Effects of PGS and PGSB on pain attenuation after burns

Typically, burn wounds cause acute pain and negative experiences, which highlights the need for pain relief in burn wound management. Owing to its analgesic, cooling, and soothing effects, borneol has been widely used in medicine and cosmetics studies 61. Given that sustained severe pain was expected to affect the metabolism and appetite of mice and, hence, their weight, we documented the weights of mice and their reactions to painful stimuli to assess pain perception. The weights of mice in the blank group decreased even after seven days of burn wound formation, with the weight loss reaching 5.6% (Figure 6E). Almost negligible weight changes were observed in the burn cream group after seven days. The weights of mice in the PGS and PGSB groups did not decrease but rather increased after only three days. The weights of mice in all groups increased in the 7-14 day period, with the fastest increase observed for the blank and burn cream groups and the highest overall weight observed in the PGSB group. MGS scoring is widely used to assess pain in mice 62. Herein, high MGS scores were found in all groups on day 0 (Figure 6F). On day 1, a decrease in the MGS scores was observed for the burn cream, PGS, and PGSB groups, especially for the PGSB group (*p* < 0.01). In contrast, the blank group demonstrated higher MGS scores. On day 3, the MGS scores for all groups decreased, most significantly in the PGSB group because of the sustained release of the analgesic borneol. On day 5, the mice showed low MGS scores because of wound recovery in all but the blank group because of the severe infection in the latter.

To further quantify the analgesic effect of PGSB, we conducted a capsaicin painting experiment on the paws of mice and determined paw lifting and licking times over 30 min (Figure S17), showing that these times were significantly lower in non-PGSB groups and thus demonstrating the excellent analgesic effect of PGSB. The analgesic effect of borneol is ascribed to TRPM8 channel activation 63. These results indicated that PGSB had best pain-relieving effect in treating infected burn wounds.

### Histological evaluation of wounds

**Figure 7:**
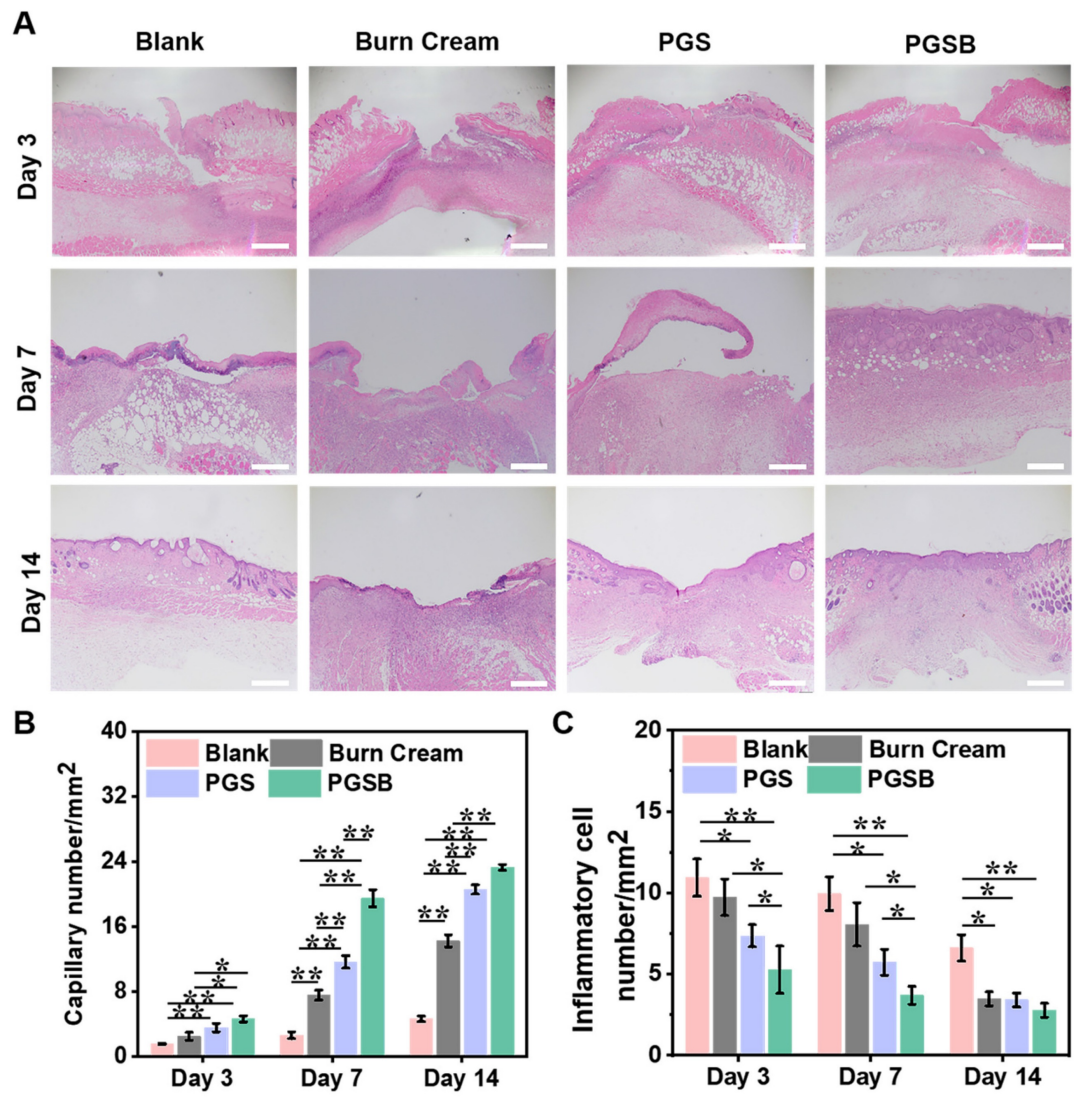
Histological staining evaluation. (A) Hematoxylin and eosin staining images of tissues obtained at days 3, 7, and 14 (scale bar = 500 μm). (B) Statistical results for capillary numbers after different treatments. (C) Statistical results for inflammatory cell numbers after different treatments. *n* = 4, *p < 0.05, **p < 0.01.

H&E staining was used to evaluate the progress of wound healing and characteristics of regenerated skin on a microscopic scale (Figures 7A and S18) Typically, inflammatory cells are recruited to the wound location because of the acute inflammatory response in the early stage of wound healing. The blank and burn cream groups demonstrated an inflammatory response even after seven days of recovery because the severe infection by *S. aureus* extended the inflammation period and delayed wound healing. In the PGS and PGSB groups, a negligible inflammatory response was observed on day 7, indicating that the hydrogels significantly alleviated the inflammatory response. After seven days of treatment, an inflammatory response was still observed in the blank control group, and a mild inflammatory response was observed in the burn cream group. In contrast, the wounds treated with PGS and PGSB moved to the proliferation stage, and numerous capillaries were formed in the PGSB group. In addition, we semi-quantitatively estimated the numbers of capillaries and inflammatory cells in tissue slices after treatment (Figures 7B-C). Capillary formation is considered advantageous for wound healing, as capillaries can fill the wound space and provide a basis for vascularization. On day 7, the PGSB group showed the highest capillary density of 19.5 mm^-2^, which was 2.6-fold higher than that in the burn cream group (*p* < 0.01). The PGS group displayed a capillary density of 11.7 mm^-2^, which was lower than that in the PGSB group but significantly exceeded that in the burn cream group (*p* < 0.01). The reverse trend was observed for the number of inflammatory cells. The number of inflammatory cells in the blank group was 1.2, 1.7, and 2.7 times higher than those in the burn cream, PGS, and PGSB groups, respectively, after seven days of treatment. After 14 days of treatment, highest number of inflammatory cells was found in the blank group, exceeding those in the burn cream, PGS, and PGSB groups 1.9-, 1.9-, and 2.4-fold, respectively. The skin tissue in the PGS and PGSB groups was almost completely regenerated, featuring successively connected morphologies, and the wounds were correspondingly remolded. The new skin tissue in the PGSB group was more mature and thicker than that in the PGS group. This result indicated that PGSB had the best therapeutic efficacy in treating *S. aureus* infected burn wounds at the microscopic histological level, confirming the superior therapeutic effects of the newly developed hydrogel platform.

### TNF-α and CD31 expression

The wound healing and tissue regeneration quality were further assessed by immunofluorescence staining methods. TNF-α and CD31 were separately selected as inflammation and angiogenesis markers, respectively. Infected wounds are believed to exhibit inflammatory responses frequently.

**Figure 8:**
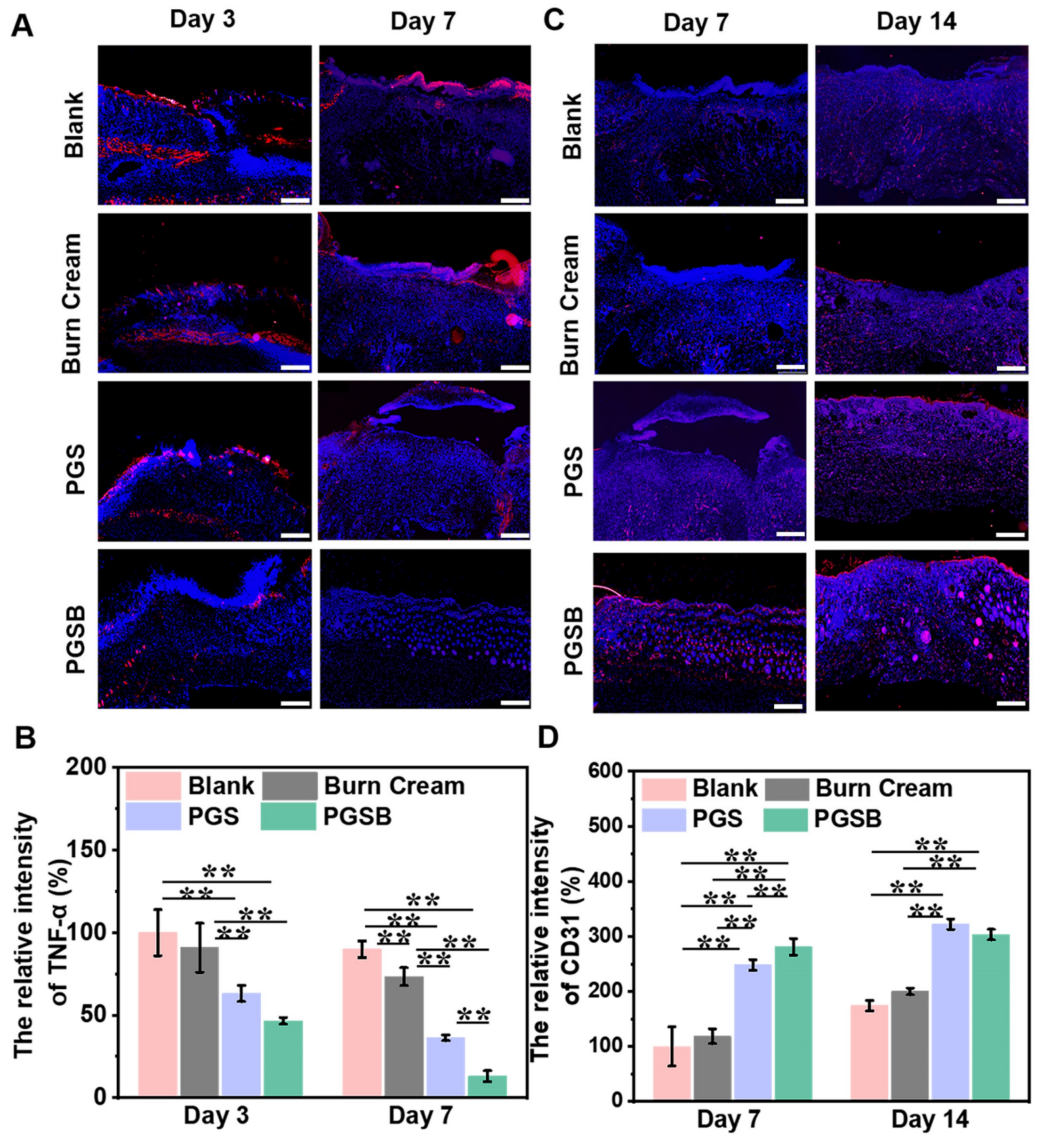
Immunofluorescence staining evaluation of TNF-α and CD31. (A) Immunofluorescence staining images of TNF-α on days 3 and 7 (scale bar = 500 μm). (B) Quantitative analysis of TNF-α on days 3 and 7. (C) Immunofluorescence staining images of CD31 on days 7 and 14 (scale bar = 500 μm). (D) Quantitative analysis of CD31 on days 7 and 14. Red fluorescence indicates positive results of expressed protein, and blue fluorescence indicates cell nuclei.* n* = 4, *p < 0.05, **p < 0.01.

The wound tissue in the blank group showed the highest expression of TNF-α at all times, while the PGS and PGSB groups presented a lower TNF-α expression on days 3 and 7 (*p* < 0.01), especially the PGSB group (Figures 8A-B and S19A). These results suggested the excellent inhibition of the inflammatory response by PGSB during the initial stage of wound repair. In addition, a notable inflammatory response was observed in the burn cream group because of the limited antibacterial and anti-inflammation activities of the burn cream. This inflammatory response was significantly higher than those in the PGS and PGSB groups (*p* < 0.01) but lower than that in the blank group (*p* < 0.01). The levels of TNF-α expression were consistent with the results of the H&E staining experiments.

Angiogenesis is a typical and critical signal of wound healing, as neovascularization at the wound site enables the transport of nutrients and oxygen and thus promotes wound healing. The expression of CD31 in the wound tissue was measured on days 7 and 14 (Figures 8C-D and S19B), with that on day 7 increasing in the order of blank < burn cream < PGS < PGSB. Similar to the abovementioned results of wound closure evaluation and H&E staining experiments, the wound tissue in the PGSB group had the highest CD31 expression, which was 2.8 and 2.4 times higher than that in the blank and burn cream groups, respectively. After 14 days, CD31 expression in the PGS and PGSB groups significantly exceeded that in the blank and burn cream groups (*p* < 0.01).

Taken together, PGSB significantly alleviated infection-induced inflammation while promoting vascularization and exhibited therapeutic effects superior to those of the commercial burn cream.

## Conclusions

This study proposes a novel pain-relieving hydrogel wound dressing (PGSB) for healing of infected burn wounds. PGSB featured crosslinks based on three physicochemical interactions, namely dynamic boronic ester bonds, Zn^2+^ coordination by PVA-GA and SA-PBA, and hydrogen bonding, and demonstrated an excellent adhesiveness, injectability, self-healing, and shape adaptability, which enabled its use in irregular dynamic wound areas. This hydrogel also showed advantageous biological functions (cooling, pain-relief, antibacterial, anti-inflammatory, antioxidant, and provascularization properties) due to the bioactive matrix and release of the borneol and Zn^2+^. In an *in vivo S. aureus* infected burn wound model, the PGSB group demonstrated the best wound closure (99.1% after 14 days), healing effects, lowest infection, and pain rates. Thus, this well-designed hydrogel platform holds promise for the healing of dynamic infected burn wounds.

## Supplementary Material

Supplementary materials and methods, figures.

## Abbreviations

CD31: platelet endothelial cell adhesion molecule
CS: chitosan
CS@BN: chitosan-coated borneol
DPPH: 1,1-diphenyl-2-picrylhydrazyl
DI: deionized
DLS: dynamic light scattering
GA: gallic acid
H&E: hematoxylin and eosin
IL-6: interleukin 6
MGS: mouse grimace scale
NMR: nuclear magnetic resonance
OD: optical density
PBA: 3-aminobenzeneboronic acid
PBS: phosphate-buffered saline
PVA: polyvinyl alcohol
PGS: hydrogel containing gallic acid-modified polyvinyl alcohol, Zn^2+^, and 3-aminobenzeneboronic acid-modified sodium alginate
PGSB: hydrogel containing gallic acid-modified polyvinyl alcohol, Zn^2+^, 3-aminobenzeneboronic acid-modified sodium alginate, and chitosan-coated borneol nanoparticles
RT-PCR: reverse transcription polymerase chain reaction
ROS: reactive oxygen species
SA: sodium alginate
SEM: scanning electron microscopy
TEM: Transmission electron microscopy
TNF-α: tumor necrosis factor α
VC: ascorbic acid
