# Supplementary material for: Multiple crosslinked, self-healing, and shape-adaptable hydrogel laden with pain-relieving chitosan@borneol nanoparticles for infected burn wound healing
Source: Theranostics. 2025 Jan 2;15(4):1439–55. doi: 10.7150/thno.102569 (PMC11729554; doi:10.7150/thno.102569)
Supplement: Supplementary file 1 — Supplementary materials and methods, figures. [file thnov15p1439s1.pdf]

Supporting material

Multiple crosslinked, self-healing, and shape-adaptable hydrogel laden with pain-relieving chitosan@borneol nanoparticles for infected burn wound healing

Zexing Deng [a, b, 1], Yi Guo [c, 1], Xuefeng Wang [a, d, 1], Jiajie Song [a], Guan Yang [a], Litong Shen [b], Yuheng Wang [a, *], Xin Zhao [e, *], Baolin Guo [e, f, *], Wen Wang [a, *]

[a] *Department of Radiology, Functional and Molecular Imaging Key Lab of Shaanxi Province, Tangdu Hospital, Air Force Medical University, Xi'an, 710038, Shaanxi, China*

[b] *College of Materials Science and Engineering, Xi'an University of Science and Technology, Xi'an, 710054, China*

[c] *Shaanxi Key Laboratory of Brain Disorders, Institute of Basic and Translational Medicine, Xi'an Medical University, Xi'an, 710021, China*

[d] *Pilot Selection Bureau of PLA Air Force, Beijing, 100195, China*

[e] *State Key Laboratory for Mechanical Behavior of Materials, and Frontier Institute of Science and Technology, Xi'an Jiaotong University, Xi'an, 710049, China*

[f] *Key Laboratory of Shaanxi Province for Craniofacial Precision Medicine Research, College of Stomatology, Xi'an Jiaotong University, Xi'an, 710049, China*

[1] Zexing Deng, Yi Guo, and Xuefeng Wang contributed equally to this work.

Corresponding authors: Wen Wang, email: wangwen@fmmu.edu.cn; Baolin Guo, email: baoling@mail.xjtu.edu.cn; Xin Zhao, email: zhaoxinbio@mail.xjtu.edu.cn; Yuheng Wang, email: 13366807682@163.com.

Materials

Acetic acid, 4-dimethylaminopyridine (DMAP), dichloromethane (CH_2Cl_2) and hydrochloric acid (HCl) were purchased from J & K. Chitosan (≥ 95% deacetylated with a viscosity of 100~200 mPa·s), 2-morpholinoethanesulphonic acid (MES) buffer, 4-(4,6-dimethoxy-1,3,5-triazin-2-yl)-4-methyl-morpholinium chloride (DMTMM), sodium alginate (SA) (M/G = 1:1 with a viscosity of 100~200 mPa·s), and 3-aminobenzeneboronic acid (PBA) were provided by Macklin. Polyvinyl alcohol (PVA) with a molecular weight of 24500, borneol, and gallic acid (GA) were provided by Aladdin. All the reagents and solvents used in this study were of analytical grade.

Specific descriptions for characterization of hydrogels and CS@BN

Freeze-dried PVA-GA, SA-PBA, and PGS was characterized by Fourier-transformed infrared spectroscopy (FT-IR, Thermo Scientific Instrument, Nicolet 6700) in the range of 4000-650 cm^{-1}. (Thermo Scientific Instrument).

Proton nuclear magnetic resonance (^1H NMR, 400 MHz) spectra of PVA-GA and SA-PBA were recorded using a Bruker Ascend instrument. D_2O served as the solvent for SA-PBA and DMSO-d_6 was used for PVA-GA.

Dynamic light scattering (DLS, Malvern instrument) was used to test the particle size and distribution of CS@BN. To further verify the stability of CS@BN, the particle size and distribution were documented at different time (day 1, day 7, and day 14).

Ultraviolet-vis spectrometer (Lambda 35, PerkinElmer) was adapted to study the borneol release behavior at different pH (5.8 and 7.4). Before all, the standard curve of

borneol was tested and recorded. Then, borneol release behavior was tested at different time.

Inductively coupled plasma mass spectrometry (ICP-MS) was used to test the zinc ions release behavior at different pH (5.8 and 7.4).

The morphological structure of PGSB and CS@BN were characterized by scanning electron microscope (SEM, Zeiss, Gemini360) and transmission electron microscope (TEM, JEOL, JEM-2100F) respectively. The hydrogel sample for SEM observation was described as follows: PGSB was first frozen and followed cut into slices. Then, sliced hydrogel samples were freeze-dried for followed observation with an accelerating voltage of 15 kV. The CS@BN sample for TEM observation was described as follows: the prepared CS@BN solution was first dispersed onto the copper mesh followed by evaporation of the solution.

The rheological property, self-healing property, and injectable property of the prepared hydrogels (20 mm in diameter) were tested using a TA rheometer (DHR-2). The rheological property was performed in angular frequency sweep mod with 1% strain and a frequency range of 0.1-100 rad/s at 25 °C. Additionally, self-healing property of the prepared hydrogels (20 mm in diameter) were tested in strain step change mode, with data recorded under 1% strain or 1000% strain at 25 °C. Moreover, the shear-thinning behavior of hydrogels (20 mm in diameter) were documented by recording the viscosity changes versus shear rate at 25 °C.

The swelling behavior and degradable properties of PGS and PGSB were tested and

characterized by weighing the hydrogel (placed at PBS buffer with a pH of 7.4 and 5.8) at different time points at 37 °C. Each group was set at least 3 repeats.

The quantitative adhesion capacity of hydrogel was studied by lab-shear test with pig skin as substrates. Specifically, pig skin was first cut into 20 mm (length) * 10 mm (width) dimension followed by placing hydrogel for adhesion. Thereafter, the quantitate adhesive strength was recorded by a tensile machine (MTS Criterion tester). In addition, the visualization of adhesive capacity of hydrogel to different substrates was confirmed by adhering hydrogel to different substrates by lifting them.

The PGSB hydrogel was prepared into a round shape with a diameter of 1 cm as flexible electrode to record electrocardiogram (ECG) signal and electromyography (EMG) signal of volunteer. The PGSB hydrogel served as working electrode mounted on the left chest or left arm to record ECG signal or EMG signal, respectively. Reference electrode and ground electrode were placed on the right arm and abdomen, respectively.

Results

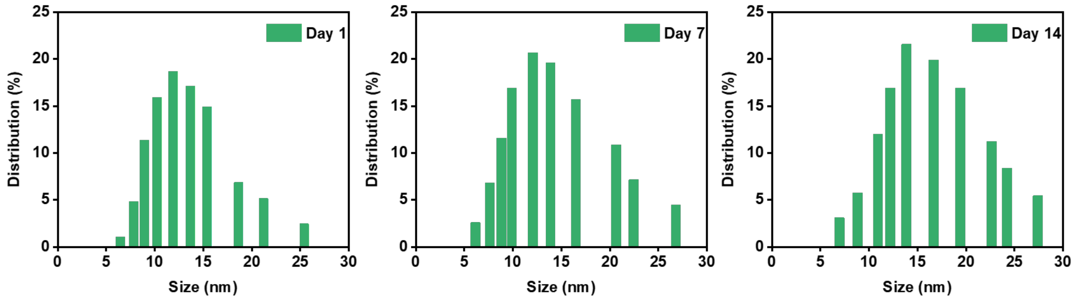

Figure S1. The CS@BN nanoparticles size distribution diagram on day 1, day 7, and day 14.

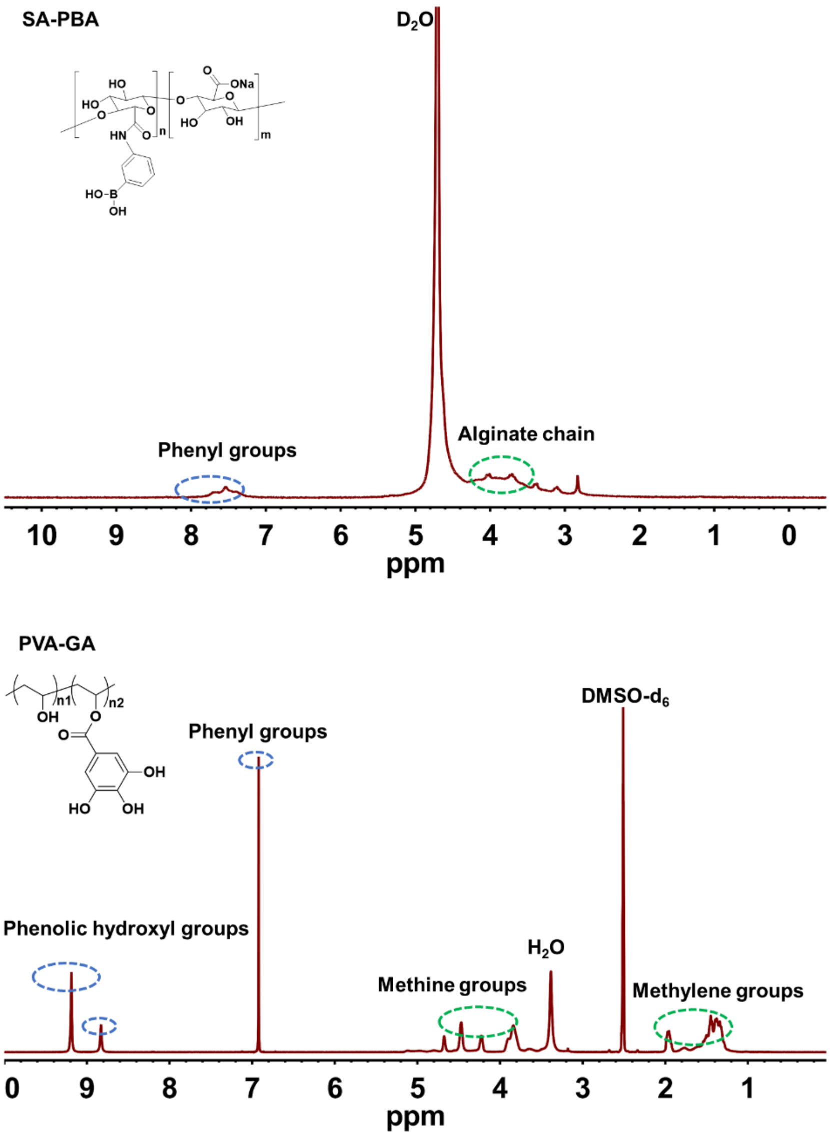

Figure S2. ^{1}H NMR spectra of SA-PBA and PVA-GA.

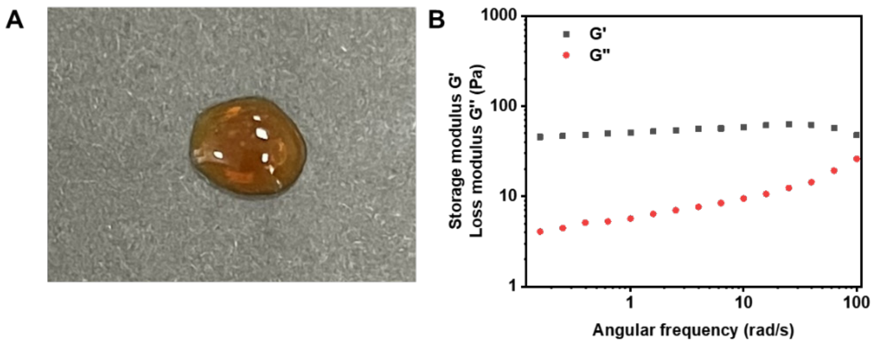

Figure S3. (A) The morphology of hydrogel without zinc ions crosslinking. (B) The rheology behavior of hydrogel without zinc ions crosslinking.

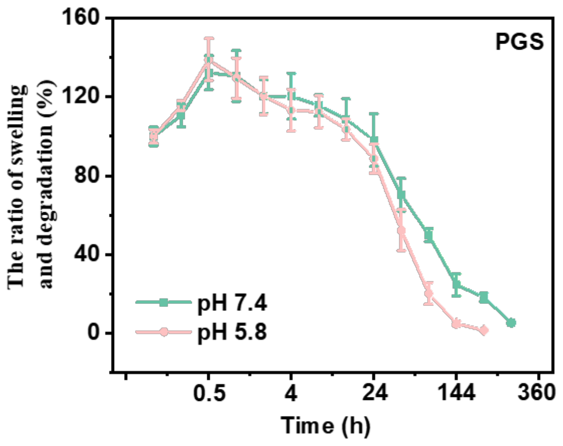

Figure S4. Swelling and degradation of PGS hydrogel at different pH (7.4 and 5.8).

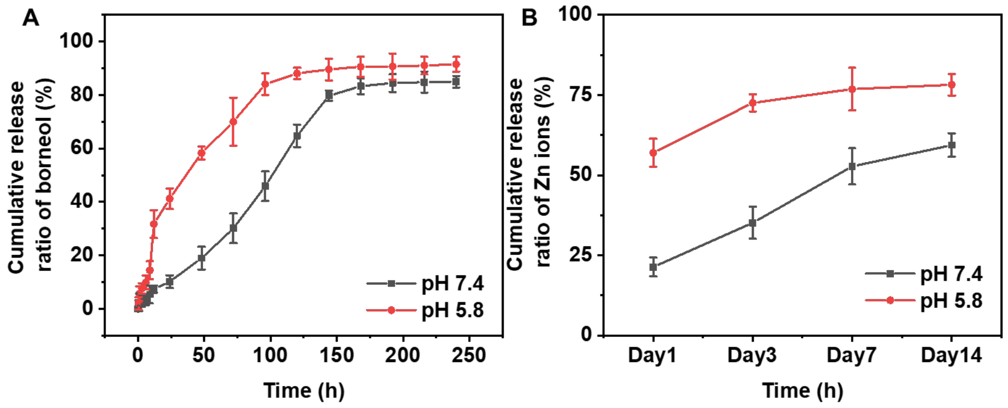

Figure S5. (A) Borneol and (B) zinc ions release curves at pH of 5.8 and 7.4.

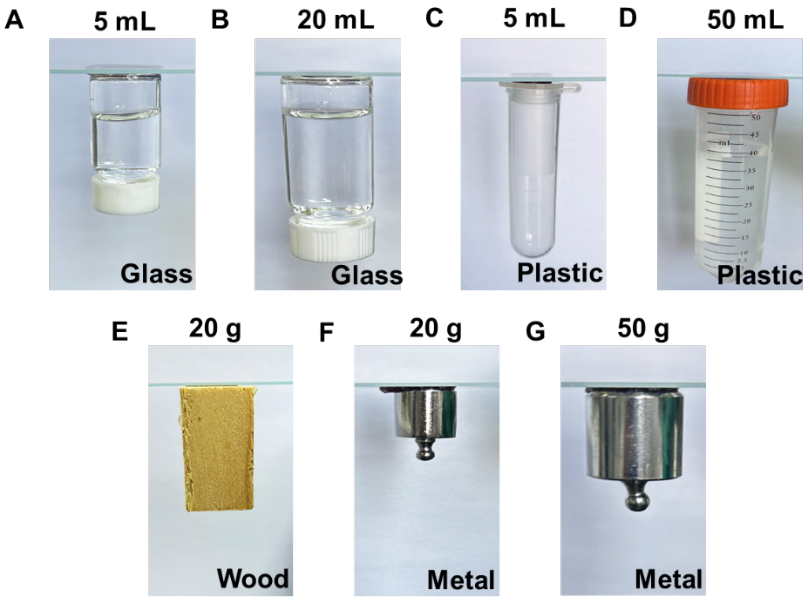

Figure S6. Demonstration of hydrogel's adhesive properties. (A-B) Hydrogel adhering

to glass bottles of 5 mL and 20 mL water. (C-D) Hydrogel adhering to plastic centrifuge tubes of 5 mL and 50 mL water. (E) Hydrogel adhering to a 20 g piece of wood. (F-G) Hydrogel adhering to metal weights of 20 g and 50 g.

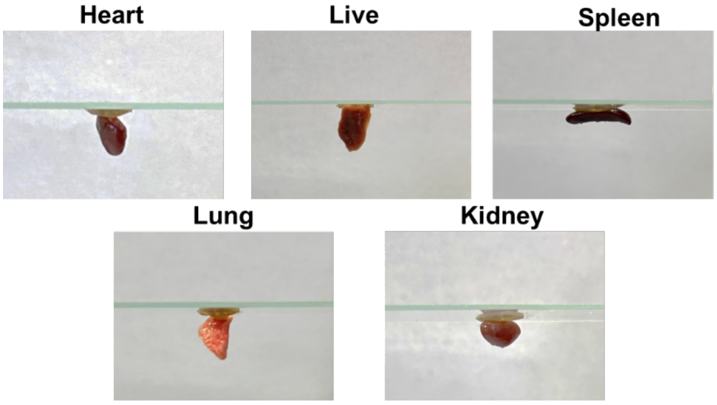

Figure S7. Demonstration of hydrogel adhesive properties on various organs.

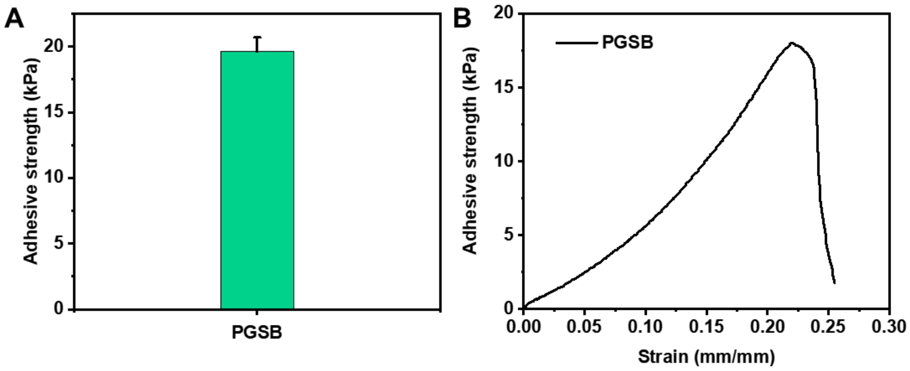

Figure S8. (A) Adhesive strength of PGSB adhering to pig skin. (B) Representative strain-adhesive strength curve based on lab-shear test.

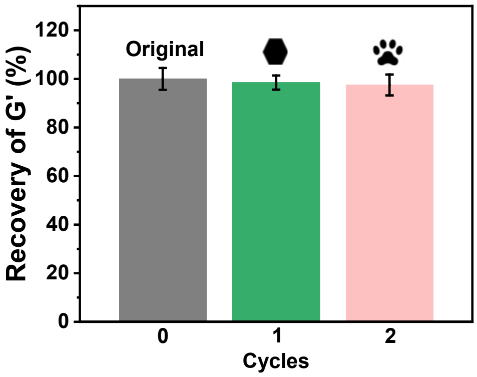

Figure S9. Recovery ratio of G' to the hexagon-shaped and cat claw–shaped hydrogels.

G' of the original hydrogel was set as 100%.

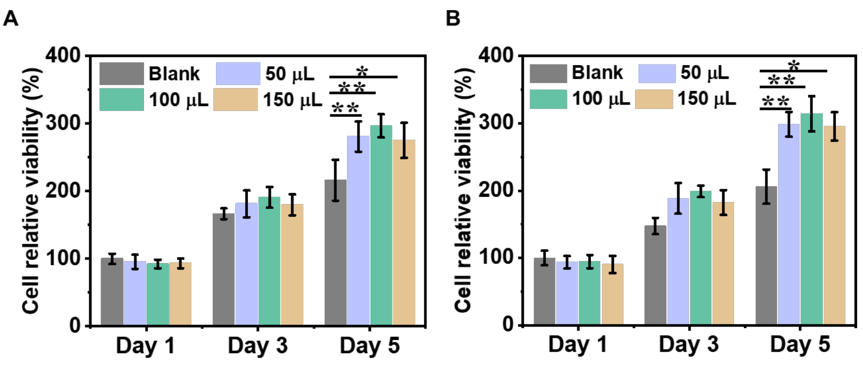

Figure S10. Cell relative viability cultured with different amounts of hydrogels. (A) PGS and (B) PGSB.

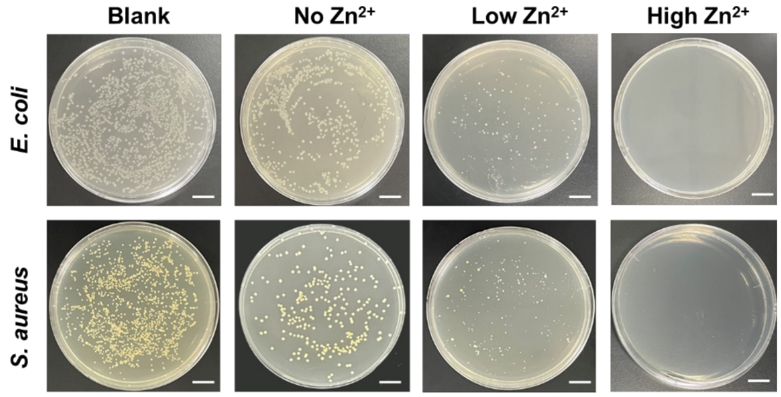

Figure S11. Antibacterial properties of hydrogels crosslinked with different concentrations of zinc ions. Scale bar = 1 cm.

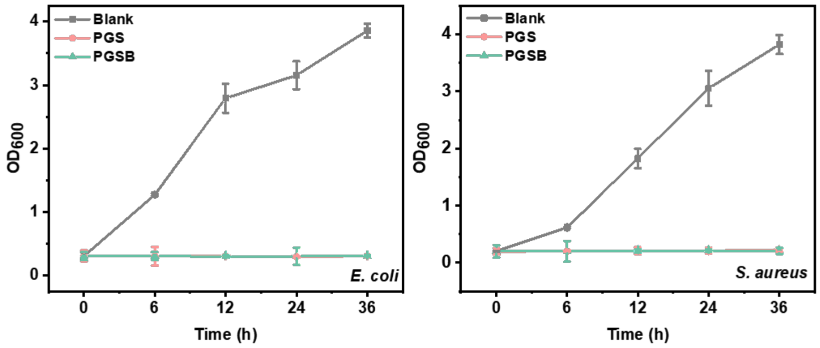

Figure S12. Antibacterial properties of PGS and PGSB hydrogels in liquid media.

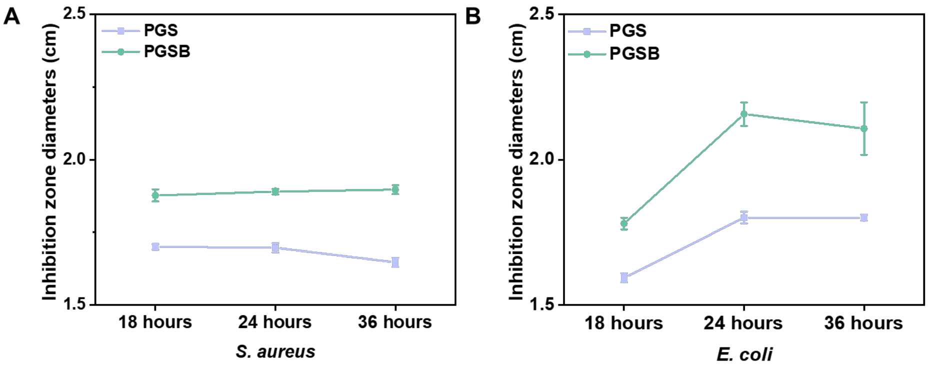

Figure S13. Statistical analysis of the diameters of inhibition zones. (A) Diameter of inhibition zones after co-culturing the hydrogel with *S. aureus* for 36 hours. (B) Diameter of inhibition zones after co-culturing the hydrogel with ampicillin-resistant *E. coli* for 36 hours.

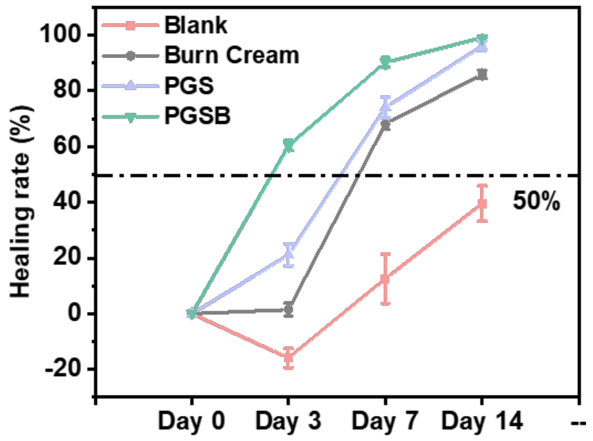

Figure S14. Proportion of wound healing on different day.

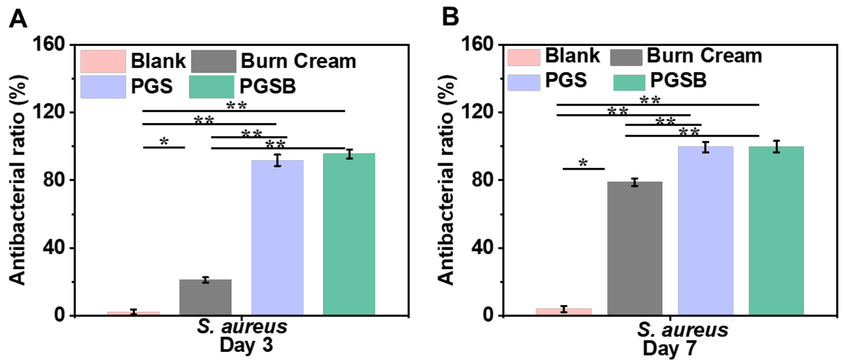

Figure S15. Statistical analysis of *in vivo* antibacterial efficacy of the hydrogel. (A) Antibacterial rate against *S. aureus* on day 3. (B) Antibacterial rate against *S. aureus* on

day 7. 'Blank' refers to the blank control, and 'Burn Cream' refers to the commercial control.

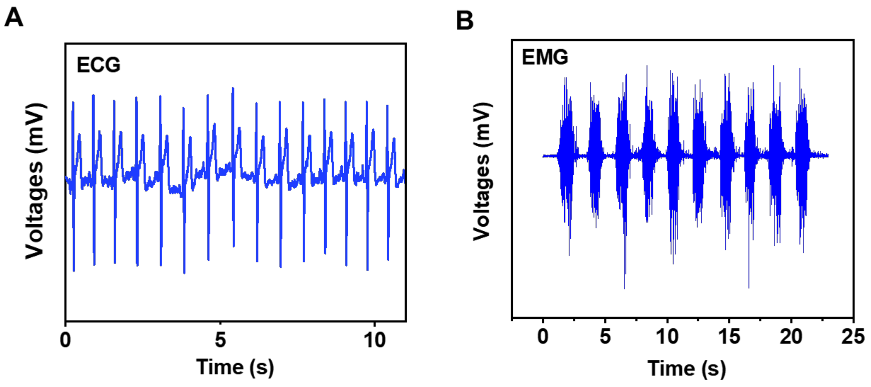

Figure S16. Electrophysiological signal tested by PGSB hydrogel. (A) Real-time ECG signal. (B) Real-time EMG signal.

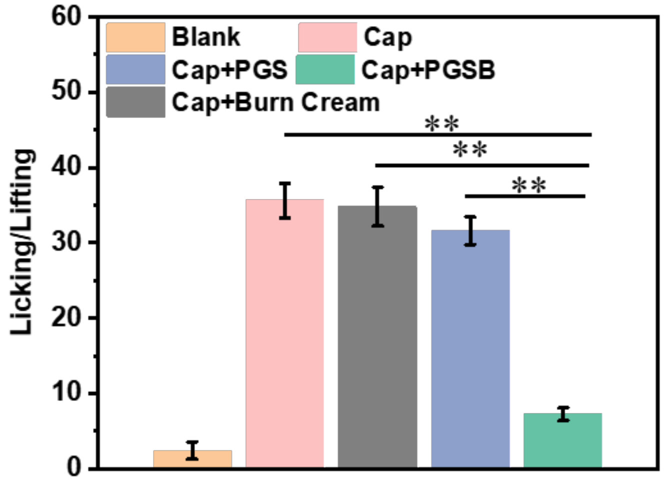

Figure S17. Paw lifting and licking times of mice for 30 minutes after painting capsaicin. The blank group is a hydrogel-free and capsaicin-free control.

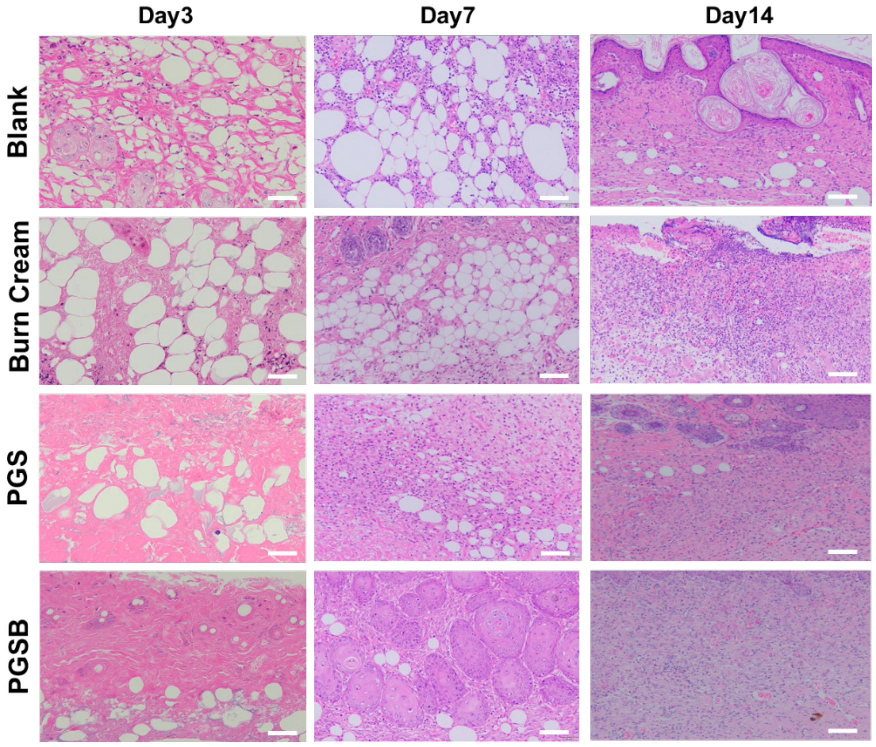

Figure S18. H&E staining results with higher magnification demonstrating the repair of infected burn skin. 'Blank' refers to the blank control, and 'Burn Cream' refers to the commercial control. Scale bar = 100 μm.

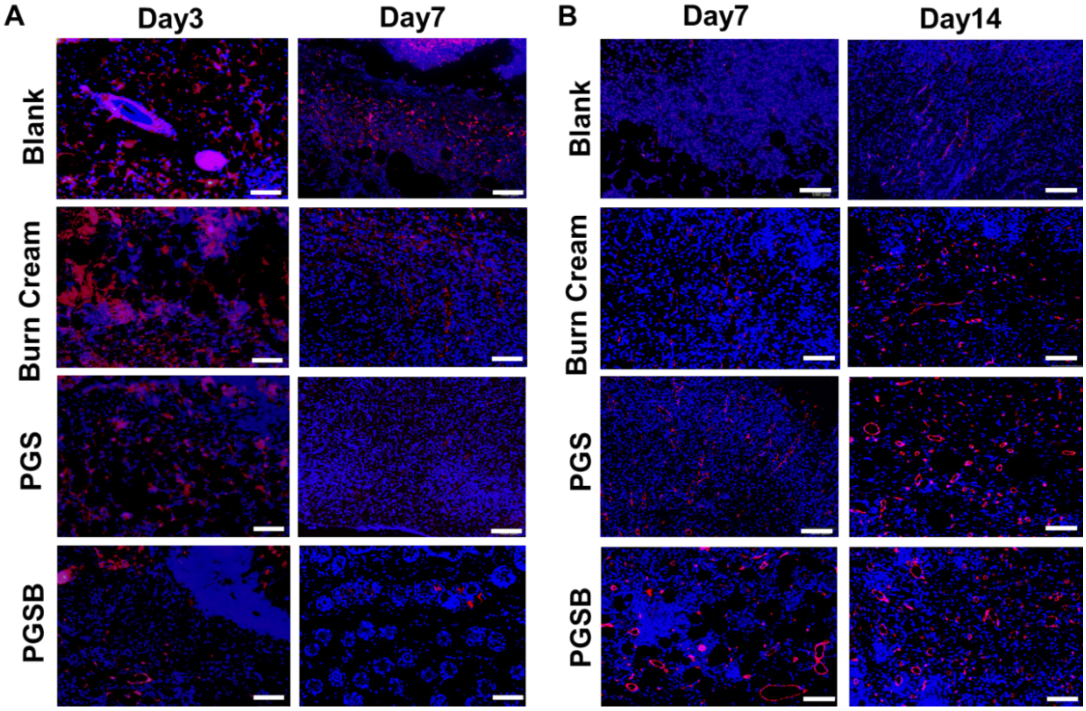

Figure S19. Immunofluorescence staining results higher magnification showing the infected burn skin repair. (A) TNF-α immunofluorescence images on days 3 and 7. (B) CD31 immunofluorescence images on days 7 and 14. Red fluorescence indicates positive results of expressed protein, and blue fluorescence indicates cell nuclei. 'Blank' refers to the blank control, and 'Burn Cream' refers to the commercial control. Scale bar = 100 μm.
